# Effectiveness and safety of fexinidazole for *gambiense* human African trypanosomiasis and exploration of adherence in outpatients: a phase 3b, prospective, open-label, non-randomised, cohort study

**DOI:** 10.1016/S2214-109X(24)00526-6

**Published:** 2025-04-24

**Authors:** Victor Kande Betu Kumeso, Christelle Perdrieu, Caroline Menétrey, Médard Ilunga Wa Kyhi, Digas Ngolo Tete, Mamadou Camara, John Tampwo, Papy Kavunga, Mariame Layba Camara, Ansoumane Kourouma, Willy Kuziena Mindele, Felix Akwaso Masa, Hélène Mahenzi, Joseph Makaya, Tim Mayala Malu, Guylain Mandula, Dieudonné Mpoyi Muamba Nzambi, Serge Luwawu Ntoya, Anne Reymondier, Wilfried Mutombo Kalonji, Bruno Scherrer, Olaf Valverde Mordt

**Affiliations:** a Ministry of Health, Kinshasa, Democratic Republic of the Congo; b Department of Research and Development, Drugs for Neglected Diseases Initiative, Geneva, Switzerland; c Human African Trypanosomiasis National Control Programme, Kinshasa, Democratic Republic of the Congo; d Human African Trypanosomiasis National Control Programme, Conakry, Guinea; e Bagata Hospital, Kwilu Province, Democratic Republic of the Congo; f Dubreka Hospital, Dubreka, Guinea; g Masi Manimba Hospital, Kwilu Province, Democratic Republic of the Congo; h Bandundu Hospital, Kwilu Province, Democratic Republic of the Congo; i Mushie Hospital, Maï Ndombe Province, Democratic Republic of the Congo; j Dipumba Hospital, Mbuji Mayi, Kasaï Oriental Province, Democratic Republic of the Congo; k Roi Baudouin Hospital, Kinshasa, Democratic Republic of the Congo; l Department of Research and Development, Drugs for Neglected Diseases initiative, Kinshasa, Democratic Republic of the Congo; m Bruno Scherrer Conseil, Saint Arnoult-en-Yvelines, France

## Abstract

**Background:**

In previous clinical trials, oral fexinidazole treatment showed a favourable safety profile, while being easily administered and effective for most adult and paediatric patients with *gambiense* human African trypanosomiasis. The aim of this open-label cohort study was to investigate the effectiveness and safety of fexinidazole in a wider population of patients and to explore the feasibility of treating patients with *gambiense* human African trypanosomiasis outside the hospital setting.

**Methods:**

In this phase 3b, prospective, open-label, non-randomised, cohort study, fexinidazole was administered orally as 600 mg tablets, in a dose regimen dependent on bodyweight. The study was conducted at eight treatment centres in the Democratic Republic of the Congo (seven of which enrolled patients) and one treatment centre in Guinea. Patients were either treated in hospital (in particular those excluded from earlier studies, such as women in their second or third trimester of pregnancy, or breastfeeding), or at home without direct medical supervision (but with the support of a caregiver). Patients were eligible for study inclusion if they met the following key criteria: aged at least 6 years; weighed at least 20 kg; had trypanosomes detected in any body fluid; had a Karnofsky performance status higher than 40%; were able to comply with the schedule of follow-up visits and with the study constraints; and were willing to undergo lumbar punctures. The primary endpoint was treatment outcome at 18 months, based on absence of parasites in lumbar puncture and blood, and overall clinical status. This study has been completed and is registered with ClinicalTrials.gov, NCT03025789.

**Findings:**

Between Nov 10, 2016, and Aug 10, 2019, 200 patients were screened, of whom 174 (87%) were included and received at least one tablet of fexinidazole: 136 patients treated in hospital and 38 treated at home. All patients but one completed treatment. At 18 months, treatment was effective in 162 (93%) of 174 patients (95% CI 88·3–96·4). No new safety signals were identified, including in the 24 women who took fexinidazole before or during pregnancy or during breastfeeding. All outpatients complied with the dosing regimen, although three (8%) of 38 completed their treatment at the hospital.

**Interpretation:**

The effectiveness and safety of fexinidazole in this wider population was similar to that described in previous clinical trials, and treatment at home seems feasible in selected patients who had the support of their caregiver.

**Funding:**

Various donors through the Drugs for Neglected Diseases initiative.

**Translation:**

For the French translation of the abstract see Supplementary Materials section.

## Introduction

Human African trypanosomiasis (sleeping sickness) is a neglected tropical disease caused by the protozoan parasites *Trypanosoma brucei gambiense* and *rhodesiense* which are transmitted by the tsetse fly. It can lead to irreversible deterioration of the nervous system and death if not treated early. *Gambiense* human African trypanosomiasis, which represents the majority of cases, is endemic in western and central Africa,^1^ with the highest number of cases found in the Democratic Republic of the Congo.^2^

Soon after infection, the parasites proliferate in the haemolymphatic system and later invade the central nervous system. Based on conclusive evidence from one pivotal study and two additional studies completed during a similar time period and in the same sites,^3^, ^4^, ^5^ fexinidazole was introduced as a first-line oral treatment for most patients, with the exception of children younger than 6 years or weighing less than 20 kg and patients with very advanced neurological disease, for whom nifurtimox and eflornithine combination therapy remains the preferred choice.

**Research in context**

**Evidence before this study**

The present study is part of a wider development programme started by the Drugs for Neglected Diseases initiative (DNDi) in 2005; therefore, most publications on fexinidazole clinical research for *gambiense* human African trypanosomiasis have been written within or around the DNDi programme. To identify other relevant publications, we did a search in PubMed and Google using the terms “human African trypanosomiasis” and “fexinidazole”, from Jan 1, 2020, until June 31, 2024. Publications in English and French were considered, including the references of those publications, and we retained those covering treatment challenges, as well as efficacy and safety of available treatments. In three previous clinical trials of the DNDi programme, oral fexinidazole was shown to be an appropriately safe, easily administered, and effective treatment for most adult and paediatric patients with *gambiense* human African trypanosomiasis. Compared with previous therapies, which required labour-intensive and inconvenient procedures, this treatment of oral fexinidazole was a major breakthrough for patients with *gambiense* human African trypanosomiasis who often live in impoverished and remote areas with poor health-care infrastructure.

**Added value of this study**

The present open-label study was innovative in two aspects. First, a cohort of selected patients were treated at home without direct medical supervision. A separate set of eligibility criteria were defined for this cohort to guarantee safety and facilitate treatment adherence. These patients knew that their adherence was to be assessed at the end of treatment. Second, patients excluded from earlier studies on fexinidazole (eg, women in their second or third trimester of pregnancy or breastfeeding women) could be included, but were to be enrolled in the cohort treated in hospital, as a precautionary measure.

**Implications of all the available evidence**

Treating patients with *gambiense* human African trypanosomiasis with fexinidazole outside the hospital setting seems feasible, even in the advanced stage of the disease, provided that patients are carefully selected and willing to be treated at home, receive clear instructions at treatment set-up, and benefit from adequate family support during treatment. No specific safety issues were detected in the women who took fexinidazole before or during pregnancy, or during breastfeeding.

A safe and easily administered oral therapy that is effective for most people with *gambiense* human African trypanosomiasis was a breakthrough. Previously available treatments required specialised health personnel for diagnosis, including lumbar puncture for cerebrospinal fluid and intravenous treatment administration. Earlier trials done in 2012 and 2016^3^, ^4^, ^5^ on fexinidazole applied restrictive eligibility criteria and treatment was administered to the patients while they were admitted to hospital at the clinical sites. The present open-label study was designed to extend the knowledge on the effectiveness and safety of fexinidazole in a wider population, under clinical conditions close to those in real life, and to explore the feasibility of treating patients with *gambiense* human African trypanosomiasis outside the hospital setting.

## Methods

### Study design and participants

This was a phase 3b, prospective, open-label, non-randomised, cohort study, conducted at eight treatment centres in the Democratic Republic of the Congo (seven of which enrolled patients) and one treatment centre in Guinea.

Patients were identified through passive screening (consultations in health structures) or active screening (by mobile teams in the endemic villages testing all the available population). Inclusion and exclusion criteria were less restrictive than in previous trials, which included patients with a Karnofsky score^6^ higher than 50, and excluded pregnant and breastfeeding women. The present study included adults and children aged six years or older and weighing 20 kg or more, with confirmed *gambiense* human African trypanosomiasis, able to swallow fexinidazole tablets with a solid meal, and with a Karnofsky score higher than 40. Parasite detection and disease staging were done in blood, lymph, and cerebrospinal fluid (table 1; appendix 2 p 3). Patients with stage 1 and intermediate *gambiense* human African trypanosomiasis were analysed together.

**Table 1:** Classification of patients according to the stage and severity of gambiense human African trypanosomiasis at inclusion

	**≤5 white blood cells per μL cerebrospinal fluid**	**6–20 white blood cells per μL cerebrospinal fluid**	**>20 white blood cells per μL cerebrospinal fluid**
Positive for parasites in the cerebrospinal fluid	Stage 2	Stage 2	Stage 2
Negative for parasites in the cerebrospinal fluid	Stage 1	Intermediate stage	Stage 2

Patients with stage 1 and intermediate stage *gambiense* human African trypanosomiasis were analysed together. Pharmacokinetic analysis was only done with inpatients because outpatient testing at day 11 was only to confirm compliance. The timepoints of blood extraction are reported in appendix 2 pp 5–8.

Patients were excluded if they had been treated for human African trypanosomiasis within 2 years or if they had severe renal or hepatic impairment, severely deteriorated general condition, or any relevant clinically active medical condition other than human African trypanosomiasis. Patients were treated for soil-transmitted helminths. Patients were also tested and, if necessary, treated for malaria.

The study population comprised two cohorts: inpatients treated in hospital, and outpatients who started treatment at home. Specific eligibility criteria were defined for outpatients to guarantee safety and facilitate treatment adherence: participants agreed to be treated outside the hospital and both outpatients and caregivers had to understand treatment administration, reside close (at most 1 h by road or boat during the treatment period) to the treatment centre, and be easily reachable during treatment. Outpatients and caregivers had to agree to be assessed on day 11 regarding treatment adherence, including a blood sample taken for analysis. Patients at any stage of the disease could be enrolled as outpatients unless neurological symptoms or medical or psychiatric contraindications for treatment at home were present.

Based on full development and reproductive toxicology studies on fexinidazole showing no risk of toxicity on fetal or postnatal development, breastfeeding and pregnant women in the second or third trimester could only be included as inpatients for precaution. Patients with a Karnofsky score of 50 or lower (requiring considerable assistance and frequent medical care) could only be enrolled as inpatients.

The study protocol was approved by the Ethics Committee of the Protestant University in the Democratic Republic of the Congo and the National Ethics Committee for Health Research in Guinea, and is available online.^7^ The protocol also received a positive opinion from the Comité de Protection des Personnes of Hôpital Necker (Paris, France). The study was done in accordance with the Declaration of Helsinki and the International Council for Harmonisation E6 (R2) Good Clinical Practice Guidelines. All participants gave written informed consent and outpatients were accompanied by their caregiver during the consent process. An independent data safety monitoring board reviewed the study data regularly.

### Procedures

Fexinidazole was administered orally as 600 mg tablets in a dose regimen dependent on bodyweight. Participants weighing more than 20 kg and less than 35 kg received 1200 mg once per day for 4 days, followed by 600 mg once per day for 6 days. Participants weighing 35 kg or more were given the full dose: 1800 mg once per day for 4 days, followed by 1200 mg once per day for 6 days. Given that food increases fexinidazole absorption, the daily dose had to be taken soon after the main meal. Temporary interruption of treatment was permitted for a maximum of 1 day, with possible addition of 1 day of treatment to compensate.^7^

Patients were observed for approximately 19 months (appendix 2 p 4). Rescue treatment was nifurtimox and eflornithine combination therapy for stage 2 *gambiense* human African trypanosomiasis and pentamidine for stage 1 *gambiense* human African trypanosomiasis. The schedule of study procedures is available in appendix 2 (pp 5–8).

### Outcomes

The primary effectiveness endpoint was fexinidazole treatment success rate 18 months after end of treatment, using a more conservative approach to define success than WHO criteria,^8^ specifically by considering all deaths (including unrelated) and patients lost to follow-up as non-successful treatment. Treatment outcome at 18 months was considered a success if the patient was alive, with no evidence of trypanosomes in any body fluid, and cerebrospinal fluid white blood cell count of 20 cells/μL or less; if lumbar puncture was missing at 18 months, but done later (eg, at 24 months) with favourable evaluation; or the patient was alive, without lumbar puncture at 18 months or later, but with lumbar puncture at 12 months with favourable evaluation (favourable evaluation is defined in the algorithms of classification in appendix 2 pp 9–11).

Success rate at 12 months was a secondary effectiveness endpoint (appendix 2 pp 12–13).

Safety assessments included adverse events reported by the patient or the investigator, signs and symptoms of human African trypanosomiasis, electrocardiograms, physical and neuropsychiatric examination, vital signs, and blood haematology and chemistry. The severity of adverse events was assessed using the National Cancer Institute Common Terminology Criteria for Adverse Events grading scale (version 4.03).

Treatment adherence and feasibility were thoroughly assessed in the outpatient cohort. At the dispensing visit (day 0), the patient and caregiver were asked about the instructions for use. In case of an incorrect answer, the study staff explained the treatment instructions again until the patient and caregiver understood. At the end of treatment visit (day 11), the questions focused on adherence to treatment instructions and packaging acceptability (appendix 2 pp 14–17).

For the pharmacokinetic assessments (only done in inpatients, because outpatient testing at day 11 was only done to confirm compliance), approximately 2 mL of whole blood was collected and deposited on filter paper using the dry blood spot technique (timepoints of blood extraction are reported in appendix 2 pp 5–8). Fexinidazole and its two active metabolites (fexinidazole sulfoxide M1 and fexinidazole sulfone M2) were quantified (timepoints in appendix 2 pp 5–8).^9^, ^10^

### Statistical analysis

Determination of the sample size was based on the primary effectiveness endpoint. The expected success rate was between 87% and 91% depending on the proportion of patients with stage 1 *gambiense* human African trypanosomiasis. The success rate for patients with stage 2 *gambiense* human African trypanosomiasis was hypothesised to be slightly below the expected success rate of 89% in the pivotal study,^3^ which had a restricted, more homogeneous population. The success rate for patients with stage 1 *gambiense* human African trypanosomiasis was expected to be close to 92%, based on a meta-analysis of 2524 patients with *gambiense* human African trypanosomiasis treated with pentamidine.^11^ A sample size of 174 patients was estimated to provide a margin of error of 5%, for an expected overall success rate of 87% and using a two-sided Wald CI of 95%. When no outcome could be applied from the decision algorithm, the primary objective was considered to have failed. We did a sensitivity analysis with multiple imputation for those cases (appendix 2 p 23).

The primary analysis was a descriptive analysis of the treatment outcome at 18 months with the conservative 95% exact Clopper–Pearson CIs in the modified intent-to-treat (mITT) population, which included all patients who took at least one tablet of fexinidazole. The same analysis was repeated in the evaluable population (excluding patients not evaluable because of missing data, and patients who died for reasons clearly unrelated to treatment effectiveness, safety, or disease evolution) and in the per-protocol population (sensitivity analysis), and also by cohort, *gambiense* human African trypanosomiasis stage, and cerebrospinal fluid white blood cell count in the mITT population (secondary analysis). The same analyses were done for the treatment outcome at 12 months as for the primary endpoint. Risk ratios were estimated using a multivariate Poisson regression to investigate the relationship between treatment failure rate at 18 months and potential covariates.

Other endpoints (safety, treatment adherence and feasibility, and pharmacokinetics) were summarised descriptively.

All summaries and statistical analyses were generated using SAS (version 9.4 or higher). This study is completed and registered with ClinicalTrials.gov (NCT03025789).

### Role of the funding source

The funder of the study had no role in the study design, data collection, data analysis, data interpretation, or writing of the clinical study report.

## Results

**Figure 1:**
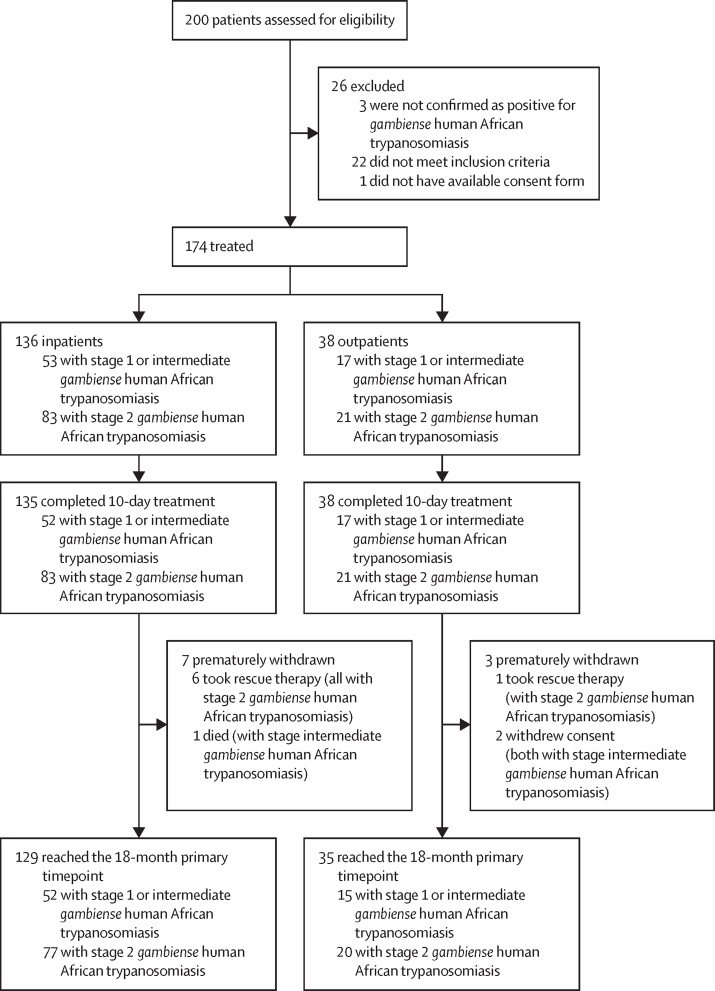
Study flowchart of complete 18-month data

Between Nov 10, 2016, and Aug 10, 2019, 200 patients were screened for the study (figure 1); 174 patients were included (136 inpatients and 38 outpatients who met specific eligibility criteria), received at least one tablet of fexinidazole, and completed treatment, except for one inpatient with stage 1 *gambiense* human African trypanosomiasis who discontinued treatment (but completed the study) after day 9 because of a serious adverse event (SAE) of anxiety. Three other inpatients discontinued treatment for 1 day, because of an adverse event of vomiting (treatment completed on day 11). Ten (6%) other patients discontinued the study: six inpatients and one outpatient used rescue therapy; two outpatients withdrew their consent; and one inpatient died (from causes unrelated to fexinidazole or *gambiense* human African trypanosomiasis).

There were 43 (25%) patients with stage 1 *gambiense* human African trypanosomiasis and 27 (15%) patients with intermediate stage *gambiense* human African trypanosomiasis who were analysed together as one subgroup (total of 70 [40%] patients), and 104 (60%) patients with advanced stage 2 *gambiense* human African trypanosomiasis (table 2). The study included more females than males. 119 (68%) patients were 15 years of age or older and 117 (67%) patients weighed 35 kg or more (and received the full dose of fexinidazole). The minimum Karnofsky score was 60 in both the inpatient and outpatient cohorts (ie, patients requiring occasional assistance, but able to care for most of their personal needs). All patients had trypanosomes detected in at least one body fluid (table 2).

**Table 2:** Baseline characteristics in the modified intent-to-treat population

			**Inpatients (n=136)**	**Outpatient (n=38)**	**Stage 1 or intermediate *gambiense* human African trypanosomiasis (n=70)**	**Stage 2 *gambiense* human African trypanosomiasis (n=104)**	**Total (n=174)**
**Demographics**							
Sex							
	Female		78 (57%)	18 (47%)	39 (56%)	57 (55%)	96 (55%)
	Male		58 (43%)	20 (53%)	31 (44%)	47 (45%)	78 (45%)
Age (years)			26·0 (15·5)	27·9 (18·0)	32·0 (17·5)	22·7 (13·9)	26·4 (16·0)
	<15 years		46 (34%)	9 (24%)	14 (20%)	41 (39%)	55 (32%)
	≥15 years		90 (66%)	29 (76%)	56 (80%)	63 (61%)	119 (68%)
Weight (kg)			42·6 (15·1)	45·2 (14·5)	46·8 (14·0)	40·7 (15·2)	43·2 (15·0)
	<35 kg		47 (35%)	10 (26%)	14 (20%)	43 (41%)	57 (33%)
	≥35 kg		89 (65%)	28 (74%)	56 (80%)	61 (59%)	117 (67%)
BMI (kg/m^2^)			18·5 (3·5)	18·6 (3·4)	19·2 (3·4)	18·0 (3·5)	18·5 (3·5)
**Diagnostic tests**							
	Positive blood or lymph test*		134 (99%)	38 (100%)	70 (100%)	102 (98%)	172 (99%)*
Screening blood tests							
	Positive CATT		133 (98%)	36 (95%)	69 (99%)	100 (96%)	169 (97%)*†
	Positive RDT		28 (21%)	7 (18%)	13 (19%)	22 (21%)	35 (20%)
Lymph examination							
	Positive lymph node aspirate		34 (25%)	12 (32%)	17 (24%)	29 (28%)	46 (26%)
Parasitological blood tests							
	Positive mAECT-BC		85 (63%)	23 (61%)	51 (73%)	57 (55%)	108 (62%)
	Positive mAECT		1 (1%)	1 (3%)	0 (0%)	2 (2%)	2 (1%)
Cerebrospinal fluid examination							
	Positive for trypanosomes		55 (40%)	14 (37%)	0 (0%)	69 (66%)	69 (40%)
	Cerebrospinal fluid white-blood-cell count		141·3 (208·0)	154·4 (230·5)	5·6 (4·0)	237·4 (232·4)	144·1 (212·5)
		≤100 cells/μL	80 (59%)	20 (53%)	70 (100%)	30 (29%)	100 (57%)
		>100 cells/μL	56 (41%)	18 (47%)	0 (0%)	74 (71%)	74 (43%)
Trypanosomes in any body fluid			136 (100%)	38 (100%)	70 (100%)	104 (100%)	174 (100%)
**Vital signs and general health**							
Systolic blood pressure (mm Hg)			102·6 (11·8)	106·3 (10·0)	105·5 (9·9)	102·1 (12·4)	103·5 (11·5)
Diastolic blood pressure (mm Hg)			66·1 (8·9)	68·2 (8·4)	67·8 (8·6)	65·7 (8·9)	66·6 (8·8)
Temperature (°C)			36·7 (0·6)	36·7 (0·6)	36·6 (0·5)	36·7 (0·6)	36·7 (0·6)
Heart rate (beats per min)			81·5 (11·2)	81·7 (9·6)	78·8 (9·4)	83·4 (11·4)	81·6 (10·9)
Respiratory rate (cycles per min)			20·4 (2·7)	19·9 (2·1)	19·8 (1·9)	20·6 (2·9)	20·3 (2·6)
Karnofsky score			86·0 (12·5)	88·9 (12·3)	90·9 (10·3)	83·8 (13·0)	86·7 (12·5)
Altered general health			47 (35%)	14 (37%)	15 (21%)	46 (44%)	61 (35%)

Data are presented as n (%) or mean (SD). BMI=body-mass index. CATT=card agglutination test for trypanosomiasis. mAECT=mini-anion exchange centrifugation technique. mAECT-BC=mini-anion exchange centrifugation technique on buffy coat. RDT=rapid diagnostic test.

* Two patients had negative CATT and all other tests in blood or lymph were negative (or not done), but their lumbar puncture was positive for trypanosomes. Blood and cerebrospinal fluid examination were done in all 174 patients, whereas lymph node aspirate was only done in 77 patients.

† Three patients did not have CATT done, but all three had positive RDT.

Swollen cervical lymph nodes were detected in 94 (54%) patients at baseline (appendix 2 pp 20–21). The most frequent signs and symptoms of *gambiense* human African trypanosomiasis were headache (124 [71%]), fever (108 [62%]), drowsiness (96 [55%]), asthenia (81 [47%]), pruritus (78 [45%]), and thinning and weight loss (76 [44%]). Other specific symptoms of central nervous system infection were reported such as insomnia (42 [24%]) and tremor (29 [17%]). No major differences between *gambiense* human African trypanosomiasis stages were observed for the most frequent unspecific symptoms, but there was a higher prevalence of several signs and symptoms, including *gambiense* human African trypanosomiasis characteristic neuropsychiatric manifestations (drowsiness), in patients with stage 2 *gambiense* human African trypanosomiasis (appendix 2 pp 16–19).

Neuropsychiatric examination results were normal for 106 (61%) of 174 patients at baseline; the proportion of those with normal results was lower for patients with stage 2 *gambiense* human African trypanosomiasis (44 [42%] of 104 patients) than those with stage 1 or intermediate *gambiense* human African trypanosomiasis (62 [89%] of 70 patients; appendix 2 pp 20–21).

There were minor differences between cohorts at baseline. The inpatient cohort had a higher proportion of females and children (table 2). Patients with stage 2 *gambiense* human African trypanosomiasis represented 83 (61%) of inpatients and 21 (55%) of outpatients. A slightly lower proportion of inpatients had a normal neuropsychiatric examination (81 [60%] *vs* 25 [66%] of outpatients) and the prevalence of the most frequent *gambiense* human African trypanosomiasis signs or symptoms was generally over 10% higher among inpatients (appendix 2 pp 16–19).

At 18 months, the primary study endpoint showed that the treatment was effective in 162 (93%) of 174 patients in the mITT population (95% CI 88·3–96·4; table 3). Sensitivity analyses excluding non-evaluable patients and patients with major protocol deviations, or imputing missing outcomes because of premature withdrawal (whatever the reason) provided success rates of 94% (88·9–96·8) in the evaluable population (n=173), 94% (89·4–97·1) in the per-protocol population (n=170), and 98% (96·6–100) in the mITT population with multiple imputation.

**Table 3:** Treatment outcome at 12 months and 18 months in the modified intent-to-treat population

	**Cohort**		Gambiense **human African trypanosomiasis stage**		**Cerebrospinal fluid white-blood-cell count**		**Total (n=174)**
	Inpatients (n=136)	Outpatients (n=38)	Stage 1 or intermediate (n=70)	Stage 2 (n=104)	≤100 cells/μL (n=100)	>100 cells/μL (n=74)	
**Response to treatment at 18 months**							
n (%)	127 (93%)	35 (92%)	67 (96%)	95 (91%)	94 (94%)	68 (92%)	162 (93%)
95% CI	87·8–96·9	78·6–98·3	88·0–99·1	84·2–96·0	87·4–97·8	83·2–97·0	88·3–96·4
**No response to treatment at 18 months**							
n (%)	9 (7%)	3 (8%)	3 (4%)	9 (9%)	6 (6%)	6 (8%)	12 (7%)
95% CI	3·1–12·2	1·7–21·4	0·9–12·0	4·0–15·8	2·2–12·6	3·0–16·8	3·6–11·7
**Response to treatment at 12 months**							
n (%)	129 (95%)	35 (92%)	67 (96%)	97 (93%)	94 (94%)	70 (95%)	164 (94%)
95% CI	89·7–97·9	78·6–98·3	88·0–99·1	86·6–97·3	87·4–97·8	86·7–98·5	89·7–97·2
**No response to treatment at 12 months**							
n (%)	7 (5%)	3 (8%)	3 (4%)	7 (7%)	6 (6%)	4 (5%)	10 (6%)
95% CI	2·1–10·3	1·7–21·4	0·9–12·0	2·7–13·4	2·2–12·6	1·5–13·3	2·8–10·3

Data regarding success and non-response to treatment are provided as the number of patients with success over non-response (percentage of patients), with Clopper-Pearson 95% CI. The data were descriptive and no formal statistical comparison was done between subgroups.

In the mITT population, 12 (7%) patients did not respond to treatment at 18 months: nine (7%) of 136 inpatients and three (8%) of 38 outpatients. Relapse was the reason for nine non-responses to treatment at 18 months. One patient had trypanosomes in the cerebrospinal fluid, one patient with a cerebrospinal fluid white-blood-cell count higher than 20 cells per μL, and seven patients received rescue medication (probable relapses). The three remaining treatment failures were due to death (one inpatient) and being lost to follow-up (two outpatients had no lumbar puncture at 12 months and 18 months and withdrew their consent 17·5 months after end of treatment). Success rates at 18 months were similar between cohorts, *gambiense* human African trypanosomiasis stages, and baseline cerebrospinal fluid white-blood-cell count subgroups (table 3). Of the 55 (32%) patients younger than 15 years, three did not respond to treatment at 18 months, corresponding to a success rate of 94·5% (95% CI 84·9–98·9). None of the tested covariates (baseline cerebrospinal fluid white-blood-cell count, adherence, and baseline symptom score) were found to be statistically significantly related to treatment failure at 18 months (appendix 2 p 23). At 12 months, treatment was effective in 164 (94%) of the 174 patients in the mITT population (95% CI 89·7–97·2), with similar success rates between cohorts or subgroups (table 3).

Clinical signs and symptoms of *gambiense* human African trypanosomiasis and abnormalities observed during physical and neurological examination decreased rapidly after fexinidazole treatment, more specifically drowsiness and insomnia (appendix 2 pp 17–22).

All 38 outpatients completed fexinidazole treatment. Although eight (21%) of 38 outpatients misunderstood initial instructions, a 100% adherence was achieved thanks to the study staff explaining the treatment again and caregivers supporting administration. There were no temporary or permanent treatment discontinuations, although three (8%) outpatients were admitted to hospital during the treatment period, where they completed the treatment. Hospital admission was due to an adverse event in two cases, and due to the investigator's decision in the other case (motivated by frequent calls from the patient). The remaining 35 outpatients completed the interview after treatment; all complied with the dosing regimen, and two (5%) were readministered fexinidazole because of vomiting within 30 min of intake, which was similar to the vomiting rate observed in inpatients (nine [7%]).

The concentrations of fexinidazole and its active metabolites, M1 (fexinidazole sulfoxide) and M2 (fexinidazole sulfone), were similar for both cohorts and for the patients who died, were lost to follow-up, or did not respond to treatment when compared with the median of the whole population (appendix 2 pp 27–31).

348 adverse events were reported in 110 (63%) of 174 patients, with no clinically relevant differences between cohorts or *gambiense* human African trypanosomiasis stages (table 4; and appendix 2 pp 24–26). The most frequently reported adverse events were vomiting (24%), headache (18%), nausea (16%), asthenia (13%), insomnia (11%), and pyrexia (11%). Most adverse events were mild or moderate, with 14 (8%) patients having at least one severe adverse event (table 5). Four severe adverse events were considered drug-related in four (2%) patients in the inpatient cohort, including anaemia, blood potassium increase, anxiety, and headache.

**Table 4:** Incidence of treatment-emergent adverse events and serious adverse events

	**Inpatients (n=136)**	**Outpatients (n=38)**	**Stage 1 or intermediate *gambiense* human African trypanosomiasis (n=70)**	**Stage 2 *gambiense* human African trypanosomiasis (n=104)**	**Total (n=174)**
Any adverse event	84 (62%) [273]	26 (68%) [75]	49 (70%) [160]	61 (59%) [188]	110 (63%) [348]
Any adverse event leading to treatment discontinuation (temporary or permanent)	4 (3%) [4]	0	2 (3%) [2]	2 (2%) [2]	4 (2%) [4]
Any adverse event leading to permanent treatment discontinuation	1 (1%) [1]	0	1 (1%) [1]	0	1 (1%) [1]
Any mild or moderate adverse event	81 (60%) [259]	25 (66%) [71]	46 (66%) [149]	60 (58%) [181]	106 (61%) [330]
Any severe adverse event	10 (7%) [14]	4 (11%) [4]	8 (11%) [11]	6 (6%) [7]	14 (8%) [18]
Any drug-related adverse event	64 (47%) [145]	18 (47%) [49]	37 (53%) [96]	45 (43%) [98]	82 (47%) [194]
Any serious adverse event	10 (7%) [13]	4 (11%) [4]	7 (10%) [9]	7 (7%) [8]	14 (8%) [17]
Any serious adverse event leading to treatment discontinuation (temporary or permanent)	1 (1%) [1]	0	1 (1%) [1]	0	1 (1%) [1]
Any serious adverse event leading to permanent treatment discontinuation	1 (1%) [1]	0	1 (1%) [1]	0	1 (1%) [1]
Any serious drug-related adverse event	1 (1%) [1]	0	1 (1%) [1]	0	1 (1%) [1]
Any serious adverse event that led to death	1 (1%) [2]	0	1 (1%) [2]	0	1 (1%) [2]

Data are presented as the n (%) [number of treatment-emergent events] in each category.

**Table 5:** All treatment-emergent adverse events from grade 3–5

		**Inpatients (n=136)**	**Outpatients (n=38)**	**Stage 1 or intermediate *gambiense* human African trypanosomiasis (N=70)**	**Stage 2 *gambiense* human African trypanosomiasis (N=104)**	**Total (n=174)**
Any severe adverse event		10 (7%) [14]	0	8 (11%) [11]	6 (6%) [7]	14 (8%) [18]
Nervous system disorders		4 (3%) [4]	0	1 (1%) [1]	3 (3%) [3]	4 (2%) [4]
	Epilepsy	2 (1%) [2]	0	1 (1%) [1]	1 (1%) [1]	2 (1%) [2]
	Headache	1 (1%) [1]	0	0	1 (1%) [1]	1 (1%) [1]
	Psychomotor hyperactivity	1 (1%) [1]	0	0	1 (1%) [1]	1 (1%) [1]
Infections and infestations		2 (1%) [2]	2 (5%) [2]	3 (4%) [3]	1 (1%) [1]	4 (2%) [4]
	Appendicitis	0	1 (3%) [1]	0	1 (1%) [1]	1 (1%) [1]
	Cholera	0	1 (3%) [1]	1 (1%) [1]	0	1 (1%) [1]
	Malaria	1 (1%) [1]	0	1 (1%) [1]	0	1 (1%) [1]
	Tuberculous pleurisy	1 (1%) [1]	0	1 (1%) [1]	0	1 (1%) [1]
Psychiatric disorders		1 (1%) [1]	2 (5%) [2]	2 (3%) [2]	1 (1%) [1]	3 (2%) [3]
	Abnormal behaviour	0	1 (3%) [1]	0	1 (1%) [1]	1 (1%) [1]
	Anxiety	1 (1%) [1]	0	1 (1%) [1]	0	1 (1%) [1]
	Confusional state	0	1 (3%) [1]	1 (1%) [1]	0	1 (1%) [1]
Blood and lymphatic system disorders		2 (1%) [2]	0	2 (3%) [2]	0	2 (1%) [2]
	Anaemia	2 (1%) [2]	0	2 (3%) [2]	0	2 (1%) [2]
Pregnancy, puerperium, and perinatal conditions		2 (1%) [2]	0	1 (1%) [1]	1 (1%) [1]	2 (1%) [2]
	Stillbirth	1 (1%) [1]	0	0	1 (1%) [1]	1 (1%) [1]
	Transverse presentation	1 (1%) [1]	0	1 (1%) [1]	0	1 (1%) [1]
Injury, poisoning, and procedural complications		1 (1%) [1]	0	0	1 (1%) [1]	1 (1%) [1]
	Uterine rupture	1 (1%) [1]	0	0	1 (1%) [1]	1 (1%) [1]
Investigations		1 (1%) [1]	0	1 (1%) [1]	0	1 (1%) [1]
	Blood potassium increased	1 (1%) [1]	0	1 (1%) [1]	0	1 (1%) [1]
Metabolism and nutrition disorders		1 (1%) [1]	0	1 (1%) [1]	0	1 (1%) [1]
	Starvation	1 (1%) [1]	0	1 (1%) [1]	0	1 (1%) [1]

Data are presented as n (%) [number of treatment-emergent events]. Dictionary used Medical Dictionary for Regulatory Activities version 19.1.

Of the 17 SAEs reported during the study, four started during fexinidazole treatment: epileptic seizures in two inpatients (one with previous history); anxiety in one inpatient who discontinued treatment (the SAE resolved about 1 month later without sequelae and was considered related to the study drug); and confusional state in one outpatient who completed fexinidazole treatment in hospital. One inpatient with a medical history of tuberculosis and alcoholism died on day 50 following two SAEs of tuberculous pleurisy and starvation that were not considered related to fexinidazole treatment. In addition, one patient died during screening, and one died after study completion.

Exposure to fexinidazole before (three patients) or during pregnancy (four patients), and during breastfeeding (17 patients), did not raise any concerns. The babies developed normally, except one who died from an SAE of neonatal infection (mother exposed to fexinidazole before pregnancy) and two who died because of anaemia in the context of severe or complicated malaria (mothers and babies exposed during breastfeeding). None of these fatal SAEs were considered related to fexinidazole.

There were no signs of liver injury and no events that could indicate potential proarrhythmic effects of the treatment (apart from one mild adverse event of palpitations). Mean QT interval corrected according to Fridericia's formula increased by about 10 ms with the maximum at day 4 during the treatment period. Clinical examination revealed a rapid improvement of *gambiense* human African trypanosomiasis signs and symptoms after 10 days of treatment, as illustrated by the decrease in the prevalence of drowsiness (from 96 [55%] to ten [6%] patients) and insomnia (from 42 [24%] to 20 [12%] patients; appendix 2 pp 17–22).

## Discussion

The present study explores the feasibility of patients with *gambiense* human African trypanosomiasis taking the 10-day fexinidazole treatment at home, without direct medical supervision. The study confirmed the effectiveness of fexinidazole in 174 patients who received 9 days or 10 days course of treatment, of whom 35 received the full course of fexinidazole treatment at home. The assessment of treatment outcome was highly conservative, with deaths regardless of the cause and loss to follow-up considered as non-response to treatment. The risk of observing false successes was low thanks to the multiple approaches to assessing outcome after treatment (clinical, parasitological, and biological), and technical supervision. Because of this conservative approach, the 12 patients who did not respond to treatment at 18 months included one death from causes unrelated to fexinidazole and two treatment failures due to patients lost to follow-up.

The success rates at 18 months by *gambiense* human African trypanosomiasis stage (95 [91%] for stage 2 and 67 [96%] for stage 1 or intermediate) were consistent with the pivotal study on fexinidazole, showing a 91% success rate in 262 patients older than 15 years with stage 2 *gambiense* human African trypanosomiasis,^3^ and with the additional study showing a 98% success rate in 230 patients older than 15 years with stage 1 or intermediate *gambiense* human African trypanosomiasis.^4^ The success rate at 18 months in the 55 (94%) patients younger than 15 years was close to that obtained in the paediatric study showing a 98·4% success rate in 125 children aged 6–14 years of any *gambiense* human African trypanosomiasis stage.^5^

The patients improved rapidly after fexinidazole treatment, as illustrated by a decrease in drowsiness and insomnia, two key markers of sleeping sickness diagnosis.^12^, ^13^ Drowsiness, and to a lower extent insomnia, were frequently observed before treatment, even in patients with stage 1 *gambiense* human African trypanosomiasis, confirming previous findings.^13^, ^14^ Early signs of sleep disturbances can be explained by the accumulation of parasites in the choroid plexus and circumventricular organs, which do not have a blood–brain barrier and are close to the neural centres that regulate sleep.^15^

The safety results are consistent with the known safety profile of fexinidazole in patients with stage 1 and 2 *gambiense* human African trypanosomiasis. The study added to the existing laboratory safety database, with a particular focus on haematology and metabolic biochemistry, including liver function. There were no new safety signals and no specific concerns in women who took fexinidazole before or during pregnancy, or during breastfeeding. Although the potential risk of embryotoxicity cannot be ruled out, this risk needs to be balanced against the risk of long-term sequelae due to congenital *gambiense* human African trypanosomiasis that was reported in babies born to mothers treated after delivery.^16^ Patients requiring considerable assistance and frequent medical care (Karnofsky score of 50) were also eligible, but none were enrolled.

No specific or additional safety issues were noted in the patients who took fexinidazole at home with clear instructions on treatment administration and a caregiver's support, and adherence to treatment turned out to be excellent in this cohort. However, these results were obtained in a small cohort of carefully selected patients who did not present any contraindication for treatment at home, had the support of a relative, and were aware of being evaluated for the treatment adherence by a blood exam on day 11. They cannot be generalised to all patients with *gambiense* human African trypanosomiasis, but they suggest that direct supervision of treatment intake by a health-care professional, as recommended by WHO,^17^ might not always be necessary, provided that the capacity of the patient to receive treatment at home is carefully assessed. The flexibility home treatment offers should contribute towards the WHO target of eliminating *T b gambiense* by 2030.

A potential selection bias cannot be ruled out because of the simultaneous recruitment for a study on acoziborole, also conducted in patients with *gambiense* human African trypanosomiasis but with more stringent eligibility criteria than the present study.^18^ At the sites shared between the two studies, patients who were not eligible for the acoziborole study (eg, pregnant and breastfeeding women) were preferentially enrolled in the present fexinidazole study, which could partly explain the higher proportion of females than males in the study population.

A performance bias might be caused by these patients being likely to modify their behaviour given that they knew they were being observed and assessed for their adherence, which limits the external validity of the results.

Fexinidazole, an easy-to-use oral drug, has an acceptable effectiveness and safety across all stages of *gambiense* human African trypanosomiasis. Further post approval pharmacovigilance is recommended due to the limited number of participants in this and previous clinical trials with fexinidazole.

### Contributors

### Equitable partnership declaration

### Data sharing

The individual patient data underlying the results of this study are available upon request to ensure adequate measures required to preserve the participants’ confidentiality can be put in place before sharing. Interested researchers may contact DNDi, commissioner of this study, for data access requests via email at CTdata@dndi.org. Researchers can also request data by completing the form available at https://www.dndi.org/category/clinical-trials/. In this form, they confirm that they will share data and results with DNDi and will publish any results as open access.

## Declaration of interests

BS reports personal fees from Drugs for Neglected Diseases initiative (DNDi) during the conduct of the study and personal fees from DNDi outside the submitted work. OVM, WMK, DNT, CP, CM, and AR report employment at DNDi. All other authors declare no competing interests.

## Supplementary Materials

French translation of the abstract

Supplementary appendix 2

Equitable Partnership Declaration
