# Supplementary material for: Effectiveness and safety of fexinidazole for gambiense human African trypanosomiasis and exploration of adherence in outpatients: a phase 3b, prospective, open-label, non-randomised, cohort study
Source: Lancet Glob Health. 2025 Apr 24;13(5):e900–9. doi: 10.1016/S2214-109X(24)00526-6 (PMC12041186; doi:10.1016/S2214-109X(24)00526-6)
Supplement: French translation of the abstract [file mmc1.pdf]

THE LANCET
Global Health

## Supplementary appendix 1

This translation in French was submitted by the authors and we reproduce it as supplied. It has not been peer reviewed. *The Lancet*'s editorial processes have only been applied to the original in English, which should serve as reference for this manuscript.

Cette traduction en français a été proposée par les auteurs et nous l'avons reproduite telle quelle. Elle n'a pas été examinée par des pairs. Les processus éditoriaux du *Lancet* n'ont été appliqués qu'à l'original en anglais et c'est cette version qui doit servir de référence pour ce manuscrit.

Supplement to: Kumeso VKB, Perdrieu C, Menétrey C, et al. Effectiveness and safety of fexinidazole for *gambiense* human African trypanosomiasis and exploration of adherence in outpatients: a phase 3b, prospective, open-label, non-randomised, cohort study. *Lancet Glob Health* 2025; **13:** e900–09.

**Supplementary materials #1**

**Summary in French**

# Étude de cohorte, ouverte, évaluant l'efficacité et la sécurité du fexinidazole chez des patients atteints de trypanosomiase humaine africaine à *Trypanosoma brucei gambiense,* explorant l'observance chez ceux traités à domicile


## Généralités

La trypanosomiase humaine africaine (THA) est une maladie tropicale négligée potentiellement mortelle. À l'heure actuelle, suite à l'introduction du fexinidazole en 2018, le choix du médicament utilisé pour traiter la THA à *T. b gambiense* (THA-g) n'est plus dépendante de la détermination du stade d'avancement de la maladie dans le système nerveux centrale par une ponction lombaire systématique. Cependant, certains malades avec des symptômes neuropsychiatriques de gravité ou les enfants de moins de 6 ans et moins de 20 kg, doivent encore être traités par les médicaments préexistants : la pentamidine chez les patients au stade précoce et l'association de nifurtimox et eflornithine (NECT) chez les patients au stade tardif. Le fexinidazole est un 5-nitroimidazole, administré par voie orale, ayant montré une activité trypanocide *in vitro* et *in vivo* contre les souches de parasites responsables de la THA. Plusieurs essais cliniques ont permis de montrer que le traitement oral au fexinidazole est sûr, facilement administrable, et efficace pour la plupart des patients (adultes et enfants) atteints de THA-g.

L'objectif de la présente étude est de fournir des informations supplémentaires, incluant des participants exclus dans des études antérieures, sur l'efficacité et la sécurité du fexinidazole et d'évaluer son utilisation dans des conditions aussi proches que possible de la "vie réelle", que ce soit en ambulatoire ou en milieu hospitalier en fonction de l'état clinique.

## Méthodes

Nous avons réalisé une étude de cohorte, multicentrique, ouverte, prospective, dans 9 centres ayant l'expérience du traitement de la THA, 8 en République Démocratique du Congo (RDC) (7 ayant effectivement recruté au moins un participant) et 1 en Guinée. Nous avons recruté une population d'adultes et d'enfants âgés d'au moins 6 ans, pesant au moins 20 kg, tous atteints de la THA-g, capables d'avaler les comprimés au fexinidazole avec un repas solide, et avec un score de Karnofsky supérieur à 40.

Les participants ont été traités à domicile ou hospitalisés selon des critères d'inclusion/exclusion prédéfinis et plus souples que dans les essais cliniques précédents. Ainsi, des patients fragiles ou à risque (comme les femmes enceintes au deuxième ou troisième trimestre de grossesse, ou les femmes allaitantes) ont pu être inclus, à condition d'être traités à l'hôpital. La cohorte des participants traités à domicile a permis d'évaluer la faisabilité du traitement en ambulatoire, sans supervision médicale directe, chez des patients atteints de THA-g. Les participants traités à domicile étaient tous assistés d'un proche qui les aidait à prendre leur traitement selon les instructions. Ils savaient que leur adhérence allait être évaluée en fin de traitement.

Tous les participants ont été traités avec le fexinidazole administré par voie orale, sous forme de comprimés de 600 mg, à prendre en une seule fois pendant le repas principal. La dose quotidienne dépendait du poids corporel. Les participants avec un poids corporel ≥20 kg et <35 kg ont reçu la dose réduite: 1200 mg par jour pendant 4 jours, suivis de 600 mg par jour pendant 6 jours. Les participants avec poids corporel ≥35 kg ont reçu la dose complète: 1800 mg par jour pendant 4 jours, suivis de 1200 mg par jour pendant 6 jours. La durée totale du traitement était donc de 10 jours. Les participants ont été suivis pendant une durée de 18 mois.

Le critère d'évaluation principal était le taux de réussite du traitement par le fexinidazole 18 mois après la fin du traitement, chez tous les participants qui ont pris au moins un comprimé de fexinidazole. Ce critère a été analysé de façon descriptive. Des analyses secondaires ont évalué le taux de réussite à 18 mois par cohorte, (externes ou hospitalisés) et par stade, et selon le taux initial de leucocytes dans le liquide céphalo-rachidien (LCR). Toutes ces analyses ont été répétées sur le taux de réussite à 12 mois. La sécurité du traitement a été évaluée par une surveillance de routine. Cette étude est terminée et enregistrée auprès de ClinicalTrials.gov sous le numéro NCT03025789.

**Résultats**

Sur les 200 patients examinés entre le 10 novembre 2016 et le 10 août 2019, 174 (87%) ont finalement été inclus et ont reçu au moins un comprimé de fexinidazole: 136 participants traités à l'hôpital et 38 participants traités à domicile. La population comprenait 43 participants au stade 1 (25%) et 27 participants au stade intermédiaire (15%), qui ont été analysés ensemble (total de 70 participants, 40%), ainsi que 104 participants au stade 2 avancé (60%).

Tous les participants ont complété le traitement, à l'exception d'un participant qui a stoppé le traitement après 9 jours à cause d'un événement indésirable grave (voir ci-dessous).

À 18 mois, le traitement s'est montré efficace pour 162 (93%) des 174 participants, avec un intervalle de confiance (IC) à 95% de 88·3-96·4. Au total, 12 échecs ont été comptabilisés à 18 mois (6·9%), 9 chez les participants hospitalisés (6·6%) and 3 chez les participants traités à domicile (7·9%). Une rechute de la THA était la raison principale des échecs à 18 mois (9 échecs au total), avec 1 participant présentant des trypanosomes dans le LCR, 1 participant avec un taux de leucocytes dans le LCR >20 cellules par µL, et 7 participants qui ont reçu le traitement de secours (considérés comme rechutes probables). Les 3 autres échecs étaient 1 participant décédé et 2 participants perdus de vue. Aucune différence dans le taux de succès à 18 mois n'a été observée selon les cohortes (hôpital versus domicile), les stades de la maladie (stade 1/intermédiaire versus stade 2), ou bien le taux initial de leucocytes dans le LCR (≤100 cellules par µL versus >100 cellules par µL).

Les 38 participants traités à domicile ont tous complété le traitement en entier. Bien que 8 participants (21%) aient initialement mal compris les instructions, une observance de 100% a pu être atteinte grâce aux efforts de l'équipe médicale et de la personne accompagnant le participant, qui ont pu clarifier toute incertitude concernant l'administration du fexinidazole.

Il n'y pas eu de discontinuation temporaire ou permanente, même si 3 participants (8%) initialement traités à domicile ont terminé leur traitement à l'hôpital. L'hospitalisation était due à un événement secondaire chez 2 participants et à la décision de l'investigateur dans le 3$^{e}$ cas (suite à des appels téléphoniques répétés du participant). Les 35 autres participants

traités à domicile ont complété l'interview post-traitement; tous ont suivi la posologie correctement, et 2 (5%) ont dû prendre la dose de fexinidazole une deuxième fois car ils ont vomi dans les 30 minutes suivant la première administration. Le taux de vomissement post-administration était similaire au taux observé chez les participants hospitalisés (9 participants, 7%). Parmi les participants hospitalisés, un participant a stoppé le traitement de façon permanente après 9 jours à cause d'un événement indésirable grave (voir ci-dessous) et 3 autres participants ont stoppé le traitement temporairement (pendant une journée) à cause de vomissements.

Aucun nouveau problème de sécurité n'a été mis en évidence. Les événements indésirables les plus fréquents étaient des vomissements (24%), des céphalées (18%), des nausées (16%), de la fatigue (13%), de l'insomnie (11%), et de la fièvre (11%). Des événements indésirables survenus pendant le traitement ont été observés chez 110 participants (63%) sur 174, sans différences notables selon la cohorte ou le stade de la maladie. La plupart des événements indésirables étaient d'intensité légère ou modérée, avec 14 participants (8%) ayant des événements indésirables d'intensité sévère. Un total de 4 événements indésirables graves ont été rapportés pendant les 10 jours de traitement au fexinidazole: des crises d'épilepsie chez 2 participants hospitalisés (dont un ayant des antécédents), anxiété chez un participant hospitalisé qui a stoppé le traitement (l'événement s'est résolu un mois plus tard, sans séquelles, et a été considéré comme lié au traitement), et un état de confusion chez un participant traité initialement à domicile et qui a terminé son traitement à l'hôpital. Un participant ayant contracté par le passé la tuberculose et avec des antécédents d'alcoolisme est décédé au jour 50 suite à deux événements indésirables graves (pleurésie tuberculeuse et inanition) qui ont été considérés sans rapport avec le fexinidazole.

L'exposition au fexinidazole avant (3 participants) ou durant la grossesse (4 participants), ainsi que durant l'allaitement (17 participants) n'a pas révélé de problèmes de sécurité. Les nourrissons se sont développés normalement, à l'exception d'un nourrisson qui est décédé suite à un événement indésirable grave d'infection néonatale (la mère avait été exposée avant la grossesse) et 2 nourrissons qui sont décédés suite à une anémie dans un contexte de malaria sévère ou compliquée (les mères et nourrissons avaient été exposés pendant l'allaitement). Ces événements indésirables ont été considérés sans rapport avec le fexinidazole.

Aucune toxicité hépatique n'a été détectée. Hormis de légères palpitations chez un participant, aucun autre événement indésirable n'a semblé indiquer un effet proarythmique du fexinidazole. Un allongement moyen de l'intervalle QT (corrigé par la formule de Fridericia) et de la fréquence cardiaque d'environ 10 ms et 9 battements par minute, respectivement, a été observé. L'examen clinique des participants a révélé une amélioration rapide de la santé des participants après 10 jours de traitement, comme démontré par la diminution de la prévalence de la somnolence (de 55% à 6%) et de l'insomnie (de 24% à 12%).

**Interprétation**

Cette étude confirme que le fexinidazole oral est une option thérapeutique de première intention, sûre et efficace contre tous les stades de la trypanosomiase africaine à *T. b. gambiense*. Dans cette étude aux critères d'inclusion plus souples que précédemment, les taux d'efficacité obtenus sont similaires à ceux des essais cliniques passés. Le traitement au fexinidazole s'est avéré aussi efficace et aussi bien toléré lorsque pris à domicile, sans supervision médicale directe, à condition de définir précisément quels patients peuvent en

bénéficier, de s’assurer du soutien d’un proche, et de fournir des instructions claires concernant l’administration du traitement. Un possible biais à considérer était que les patients et les accompagnants étaient au courant que leur adhérence allait être évaluée en fin de traitement.

**Financement**

Par l’intermédiaire de DNDi (Drugs for Neglected Diseases initiative): la Fondation Bill & Melinda Gates (BMGF); UK International Development (Grande Bretagne); le Ministère Néerlandais des Affaires Étrangères (DGIS, Pays-Bas); l'Agence Norvégienne de Coopération au Développement (Norad, Norvège); le Ministère Norvégien des Affaires Étrangères (Norvège) par sa participation au deuxième programme de Partenariat pour Etudes Cliniques entre l’Union Européenne et les Pays en Voie de Développement (EDCTP2); le Ministère Fédéral de l'Éducation et de la Recherche (BMBF) par l'intermédiaire de KfW (Allemagne); la Fondation Stavros Niarchos (SNF, Grèce); Médecins Sans Frontières International (MSF); la Direction du Développement et la Coopération (SDC, Suisse); la firme Takeda Pharmaceuticals (Japon); ainsi que d'autres fondations privées et des particuliers participant à la campagne contre la THA.
