# Supplementary material for: Effectiveness and safety of fexinidazole for gambiense human African trypanosomiasis and exploration of adherence in outpatients: a phase 3b, prospective, open-label, non-randomised, cohort study
Source: Lancet Glob Health. 2025 Apr 24;13(5):e900–9. doi: 10.1016/S2214-109X(24)00526-6 (PMC12041186; doi:10.1016/S2214-109X(24)00526-6)
Supplement: Supplementary appendix 2 [file mmc2.pdf]

THE LANCET
Global Health

Supplementary appendix 2

This appendix formed part of the original submission and has been peer reviewed. We post it as supplied by the authors.

Supplement to: Kumeso VKB, Perdrieu C, Menétrey C, et al. Effectiveness and safety of fexinidazole for *gambiense* human African trypanosomiasis and exploration of adherence in outpatients: a phase 3b, prospective, open-label, non-randomised, cohort study. *Lancet Glob Health* 2025; **13:** e900–09.

Supplementary materials #2

Table of Contents

FIGURE S1. DECISION TREE FOR THE DIAGNOSIS OF G-HAT IN THE CONTEXT OF THE STUDY 3

FIGURE S2. STUDY DESIGN 4

TABLE S3. SCHEDULE OF STUDY PROCEDURES - PATIENTS TREATED IN HOSPITAL (INPATIENT COHORT) 5

TABLE S4. SCHEDULE OF STUDY PROCEDURES - PATIENTS TREATED ON AN OUT-PATIENT BASIS (OUTPATIENT COHORT) 7

TABLE S5. CLINICAL CLASSIFICATION OF G-HAT PATIENTS AT THE VARIOUS TIME POINTS 9

FIGURE S6. ALGORITHM OF CLASSIFICATION TO CATEGORIZE PATIENTS AS SUCCESS OR FAILURE FOR THE PRIMARY EFFECTIVENESS ENDPOINT (18 MONTHS) 10

FIGURE S7. ALGORITHM OF CLASSIFICATION TO CATEGORIZE PATIENTS AS SUCCESS OR FAILURE FOR THE SECONDARY EFFECTIVENESS ENDPOINT (12 MONTHS) 12

TEXT S8. SELECTION OF OUTPATIENTS 14

TABLE S9A. PRE-TREATMENT QUESTIONNAIRE TO CHECK THAT INSTRUCTIONS FOR USE OF FEXINIDAZOLE WERE UNDERSTOOD BY OUTPATIENTS (DISPENSING VISIT, DAY 0) 15

TABLE S9B. POST-TREATMENT QUESTIONNAIRE TO ASSESS OUTPATIENTS' ADHERENCE TO TREATMENT AND PACKAGING ACCEPTABILITY (END OF TREATMENT VISIT, DAY 11) 16

TEXT S10. BASELINE CLINICAL CHARACTERISTICS ACCORDING TO G-HAT STAGE AND CHANGE IN PREVALENCE OVER TIME (TABLE S11A AND TABLE S11B) 17

TABLE S11A . PREVALENCE OF CLINICAL SIGNS AND SYMPTOMS OF G-HAT OVER TIME, BY COHORT, G-HAT STAGE, AND OVERALL IN THE MODIFIED INTENT-TO-TREAT POPULATION 18

TABLE S11B.·PHYSICAL AND NEUROLOGICAL ABNORMALITIES PRESENT IN AT LEAST 10% OF PATIENTS IN ANY SUBGROUP AT BASELINE, AND CHANGE IN PREVALENCE OVER TIME BY COHORT, G-HAT STAGE, AND OVERALL IN THE MODIFIED INTENT-TO-TREAT POPULATION 21

TABLE S12. RELATIONSHIP BETWEEN FAILURE RATE AT 18 MONTHS AND POTENTIAL PREDICTORS: RESULTS OF THE POISSON REGRESSION MODEL AND GENERALIZED ADDITIVE MODELS (SPLINE) 23

TABLE S13. ALL TREATMENT-EMERGENT ADVERSE EVENTS BY SYSTEM ORGAN CLASS AND PREFERRED TERM IN THE MODIFIED INTENT-TO-TREAT POPULATION 24

FIGURE S14. DISTRIBUTION OF FEXINIDAZOLE CONCENTRATION BY VISIT FOR IN/OUT-PATIENTS IN THE PK POPULATION 27

FIGURE S15. DISTRIBUTION OF M1 CONCENTRATION BY VISIT FOR IN/OUT-PATIENTS IN THE PK POPULATION 28

FIGURE S16. DISTRIBUTION OF M2 CONCENTRATION BY VISIT FOR IN/OUT-PATIENTS IN THE PK POPULATION 29

FIGURE S17. CONCENTRATIONS OF FEXINIDAZOLE, M1, AND M2: COMPARISON OF PATIENTS WITH G-HAT RELAPSE TO THE MEDIAN, 5TH AND 95TH PERCENTILES OF ALL PATIENTS IN THE PK POPULATION 30

Figure S1. Decision tree for the diagnosis of g-HAT in the context of the study

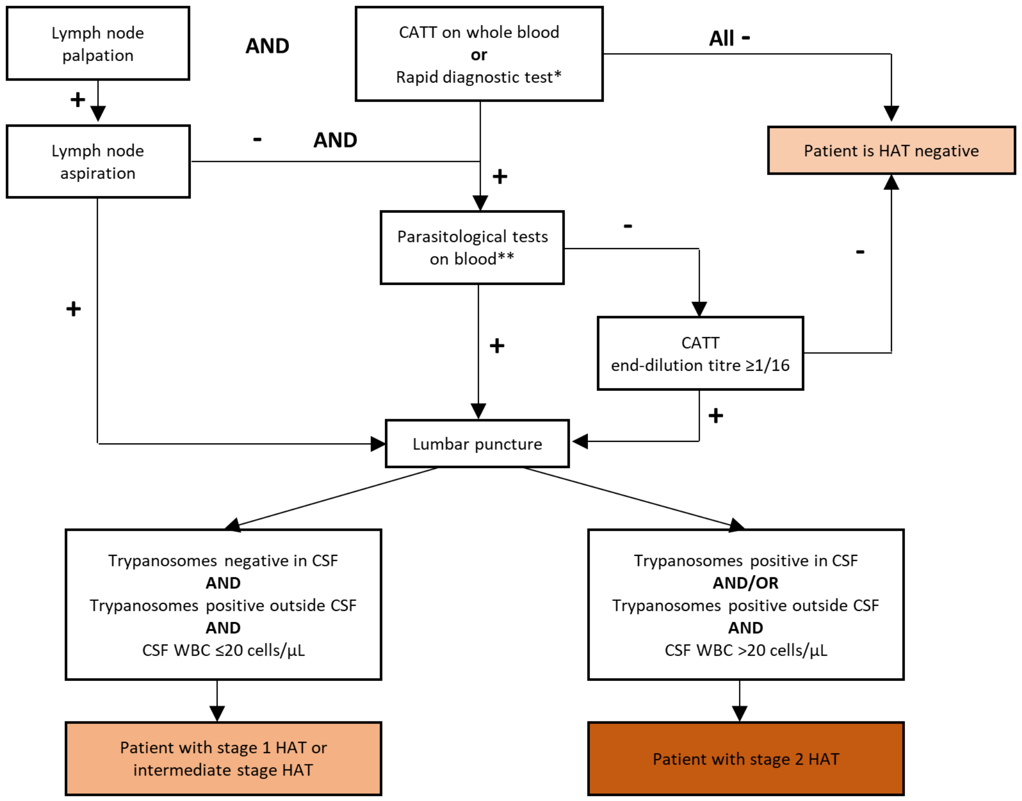

* Preferably CATT for active screening and rapid diagnostic test for passive screening.
** Preferably in the following order: mAECT-BC (passive); mAECT (active); CTC/Woo test; thick blood smear.
CATT=card agglutination test for trypanosomiasis; CSF=cerebrospinal fluid; CTC=capillary tube centrifugation; g-HAT=human African trypanosomiasis due to *T. b. gambiense*; HAT=human African trypanosomiasis; mAECT=mini-anion exchange centrifugation technique; mAECT-BC=mini-anion exchange centrifugation technique on buffy coat; WBC=white blood cell (count).

Figure S2. Study design

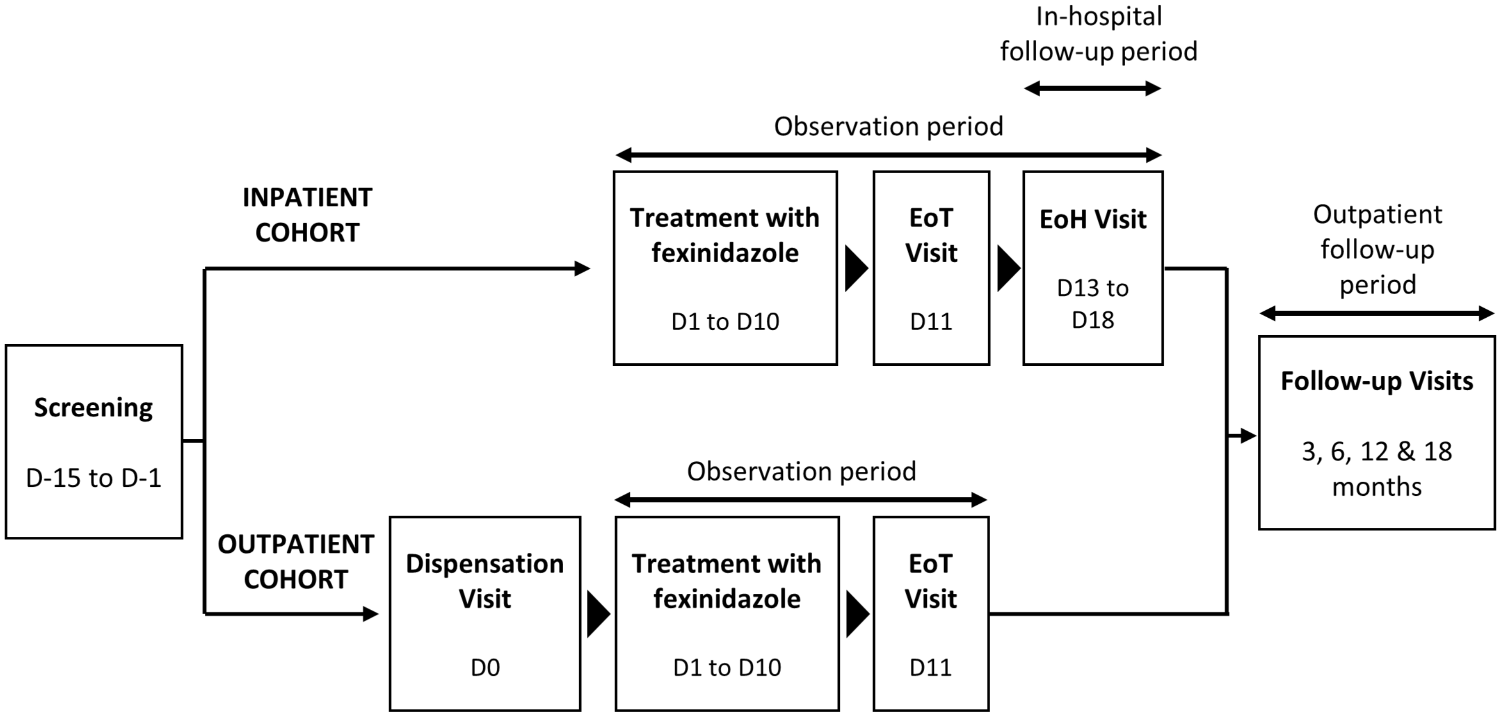

Each patient's participation in the study lasted for approximately 19 months, including the screening period (1 to 15 days), the treatment period (10 days from day 1 to day 10) and the follow-up period (18 months).
For both cohorts, the first intake of fexinidazole was scheduled at day 1 and the following study visits were scheduled at the investigational centre: day 11 (EoT Visit at end of treatment), months 3, 6, 12, and month 18 (primary effectiveness timepoint).
The observation period extended until the EoH Visit for the inpatient cohort (visit performed between day 13 and day 18) and until the EoT Visit at day 11 for the outpatient cohort. Patients treated on an out-patient basis who required hospitalisation, for whatever reason, underwent the same procedures as patients initially treated in hospital, up to the end of hospitalisation.
D=day; EoH=End of Hospitalisation; EoT=End of Treatment.

Table S3. Schedule of study procedures - Patients treated in hospital (inpatient cohort)

**Protocol-planned procedures and forms to be completed**	**Screening**	**Treatment Period**										**In-hospital Follow-up**			**Outpatient Follow-up**
Time point	D-15 to D-1	D1	D2	D3	D4	D5	D6	D7	D8	D9	D10	D11 (EoT)	D12	D13-D18 (EoH)	3, 6, 12 & 18 months
RDT or CATT	X														
Detection of parasite in blood and/or lymph	X											X			X
Informed consent (before any additional medicines or study-specific procedures)	X														
Lumbar puncture (parasite and WBCs in CSF)	X														X[a]
Pretreatment of helminthiasis[b]	X														
RDT and/or thick blood smear for diagnosis of malaria and, if necessary, treatment[b]	X														
Karnofsky performance status	X											X			X
Inclusion and exclusion criteria	X														
Demographic data	X														
Medical history	X														
Signs and symptoms of g-HAT	X											X			X
Vital signs[c]	X								X[d]			X		X	X
Physical and neurological examinations	X								X[d]			X		X	X
Haematology and biochemistry	X											X			X[e]
Urine analysis	X											X			
Urine pregnancy test	X (D-1)											X			X[f]
Triplicate ECG	X (D-1)				X D4 H4	X D4 H23						X			
IMP administration		X	X	X	X	X	X	X	X	X	X				
Blood sampling for PK analyses (specific patient groups)									X H3.15	X H3	X H3 H7.15	X D10 H24	X D10 H48		

**Protocol-planned procedures and forms to be completed**	**Screening**	**Treatment Period**										**In-hospital Follow-up**			**Outpatient Follow-up**
Time point	D-15 to D-1	D1	D2	D3	D4	D5	D6	D7	D8	D9	D10	D11 (EoT)	D12	D13-D18 (EoH)	3, 6, 12 & 18 months
AE collection	X[g]	X	X	X	X	X	X	X	X	X	X	X	X	X	X[h]
SAE collection from signature of consent form to last study visit	X	X	X	X	X	X	X	X	X	X	X	X	X	X	X
Collection of concomitant medications	X	X	X	X	X	X	X	X	X	X	X	X	X	X	

a At 3 months, only if indicated by patient's clinical status.
b Treatment according to national guidelines.
c Temperature, blood pressure, heart rate and respiratory frequency.
d Only if indicated by patient's clinical status.
e At 3 and 6 months. At 12 and 18 months only if indicated by patient's clinical status.
f At 3 and 6 months only.
g If considered related to study participation.
h If reasonable possibility of relationship to treatment.

AE=adverse event; CATT=card agglutination test for trypanosomiasis; CSF=cerebrospinal fluid; D=Day; ECG=electrocardiogram; EoT=End of Treatment; EoH=End of Hospitalisation; g-HAT=human African trypanosomiasis due to *T. b. gambiense*; H=hour; IMP=investigational medicinal product; PK=pharmacokinetic; RDT=rapid diagnostic test; SAE=serious adverse event; WBC=white blood cell.

Table S4. Schedule of study procedures - Patients treated on an out-patient basis (outpatient cohort)

**Protocol-planned procedures and forms to be completed**	**Screening**	**Dispensing**	**Treatment Period**										**Outpatient Follow-up**	
Time point	D-15 to D-1	D0	D1	D2	D3	D4	D5	D6	D7	D8	D9	D10	D11 (EoT)	3, 6, 12, & 18 months
RDT or CATT	X													
Detection of parasite in blood and/or lymph	X												X	X
Informed consent (before any additional medicines or study-specific procedures)	X													
Lumbar puncture (parasite and WBCs in CSF)	X													X[a]
Pretreatment of helminthiasis[b]	X													
RDT and/or thick blood smear for diagnosis of malaria and, if necessary, treatment[b]	X													
Karnofsky performance status	X												X	X
Inclusion and exclusion criteria	X													
Demographic data	X													
Medical history	X													
Signs and symptoms of g-HAT	X												X	X
Vital signs[c]	X												X	X
Physical and neurological examinations	X												X	X
Haematology and biochemistry	X												X	X[d]
Urine analysis	X												X	
Urine pregnancy test	X (D-1)												X	X[e]
Triplicate ECG	X (D-1)												X	
IMP dispensing / Discharge from hospital		X												
IMP administration			X	X	X	X	X	X	X	X	X	X		
Blood sampling for PK analyses													X D10H24[f]	

Protocol-planned procedures and forms to be completed	Screening	Dispensing	Treatment Period										Outpatient Follow-up	
Time point	D-15 to D-1	D0	D1	D2	D3	D4	D5	D6	D7	D8	D9	D10	D11 (EoT)	3, 6, 12, & 18 months
Understanding of instructions for treatment (questionnaire)		X												
Treatment compliance (interview)													X	
Acceptability of packaging (questionnaire)													X	
AE collection	X[g]												X[h]	X[i]
SAE collection from signature of consent form to last study visit	X	X	X	X	X	X	X	X	X	X	X	X	X	X
Collection of concomitant medications	X												X[h]	

a At 3 months, only if indicated by patient's clinical status.
b Treatment according to national guidelines.
c Temperature, blood pressure, heart rate and respiratory frequency.
d Only if indicated by patient's clinical status.
e At 3 and 6 months only.
f Additional PK sampling was performed between Day 1 and Day 11 if the patient returned to the site for an unscheduled visit.
g If considered related to study participation.
h Interview for the collection of AEs observed by the patient during the treatment period and collection of concomitant medications.
i If reasonable possibility of relationship to treatment.

AE=adverse event; CATT=card agglutination test for trypanosomiasis; CSF=cerebrospinal fluid; D=Day; ECG=electrocardiogram; EoT=End of Treatment; g-HAT=human African trypanosomiasis due to *T. b. gambiense*; H=hour; IMP=investigational medicinal product; PK=pharmacokinetic; RDT=rapid diagnostic test; SAE=serious adverse event; WBC=white blood cell.

Table S5. Clinical classification of g-HAT patients at the planned time points

Visit (timing)	Favourable outcome	Uncertain outcome	Probable relapse	Proven relapse
24 hours after EoT	• Patient alive with no evidence of trypanosomes in any body fluid			• Evidence of trypanosomes in any body fluid
3 months (±1 week)	• Patient alive with no evidence of trypanosomes in any body fluid (no lumbar puncture at 3 months unless Investigator suspects relapse)	• Any reason prompting the Investigator to request an additional follow-up visit	• Neurological signs or symptoms leading to use of rescue treatment	• Evidence of trypanosomes in any body fluid
6 months (±1 week)	• Patient alive with no evidence of trypanosomes in any body fluid and CSF WBC ≤20 cells/µL	• CSF WBC between 20 and 50 cells/µL and additional visit requested within 1-3 months<br>• Any reason prompting the Investigator to request an additional follow-up visit	• CSF WBC ≥50 cells/µL<br>• Neurological signs or symptoms leading to use of rescue treatment	• Evidence of trypanosomes in any body fluid
12 months (±4 weeks)	• Patient alive with no evidence of trypanosomes in any body fluid and CSF WBC ≤20 cells/µL<br>• CSF WBC between 20 and 50 cells/µL, and lower as compared to prior value(s)	• CSF WBC >20 cells/µL and nonsignificant increase from a clinical standpoint in relation to prior value(s)<br>• Any reason prompting the Investigator to request an additional follow-up visit	• CSF WBC >20 cells/µL and significant increase from a clinical standpoint in relation to prior value(s)<br>• Neurological signs or symptoms leading to use of rescue treatment	• Evidence of trypanosomes in any body fluid
18 months (±4 weeks)	• Patient alive with no evidence of trypanosomes in any body fluid and CSF WBC ≤20 cells/µL<br>• Patient with no signs of g-HAT and who refuses to undergo lumbar puncture and who, in the opinion of the Investigator does not require rescue treatment or an additional follow-up visit	• Any reason prompting the Investigator to request an additional follow-up visit	• CSF WBC >20 cells/µL<br>• Neurological signs or symptoms leading to use of rescue treatment	• Evidence of trypanosomes in any body fluid

CSF=cerebrospinal fluid; EoT=End of Treatment; g-HAT=human African trypanosomiasis due to *T. b. gambiense;* M=months; WBC=white blood cell.

Figure S6. Algorithm of classification to categorize patients as success or failure for the primary effectiveness endpoint (18 months)

**Derivation algorithm for stage 1 and intermediate stage (Month 18)**	**Derivation algorithm for stage 2 (Month 18)**

Patient is dead (any reason of death and any time between drug intake and M18)
⇒ YES ⇒ ***Failure (stop)***
⇓
NO (patient alive)
⇓
Patient requires rescue medication for HAT at M18 or required it before (between drug intake and M18)
⇒ YES ⇒ *(definitive)* ***Failure* (stop)**
⇓
NO rescue medication so far
⇓
Evidence of trypanosomes in any body fluid between drug intake and M18 visit
⇒ YES ⇒ *(definitive)* ***Failure* (stop)**
⇓
NO observed trypanosomes
⇓
Patient Lost to follow-up at M18 (no survival information at M18 and later)
⇒ YES ⇒ ***Failure* (Stop)**
⇓
NO (the patient is not lost to follow-up)
⇓
Non-haemorrhagic lumbar puncture at M18 and WBC in CSF at M18 >20 cells
⇒ YES ⇒ ***Failure* (stop)**
⇓
NO (WBC in CSF at M18 ≤20 cells or no reliable data concerning WBC in CSF at M18)
⇓
Non-haemorrhagic lumbar puncture at M18 and WBC in CSF ≤20 cells
⇒YES ⇒ ***Success* (stop)**
⇓
NO (haemorrhagic CSF sample or no lumbar puncture at M18 for any reason)
⇓
No lumbar puncture at M18 or no reliable count of WBC in CSF at M18 but reliable number of WBC in CSF reported later (M24 or other additional visit)
⇒ YES ⇒ **WBC in CSF >20** ⇒ ***Failure* (Stop)**
⇒ YES ⇒ **WBC in CSF ≤20** ⇒ ***Success* (Stop)**
⇓
NO (No lumbar puncture at M18 and no later reliable count of WBC in CSF)

⇓<br><br>⇓<br>**Patient has clinical signs or symptoms at M18 evoking a failure**<br>⇒ YES ⇒ ***Failure* (stop)**	⇓<br>**Earlier (before M18) unfavourable outcome: WBC in CSF >50 at M6 or WBC in CSF >20 at M12 or increasing between M6 and M12**<br>⇒ YES ⇒ ***Failure* (stop)**<br>⇓<br>NO (no information or no earlier unfavourable assessment)<br>⇓<br>**Patient has clinical signs or symptoms at M18 evoking a failure**<br>⇒ YES ⇒ ***Failure* (stop)**

**Derivation algorithm for stage 1 and intermediate stage (Month 18)**	**Derivation algorithm for stage 2 (Month 18)**
⇓<br>NO (no clinical signs or symptoms evoking a relapse at M18)<br>⇓<br>**WBC in CSF ≤20 at M12 or absence of lumbar puncture at M12 and no sign and symptoms at M18 evoking a relapse**:<br>⇒ YES ⇒ ***Success*** (stop)<br>⇓<br>NO (at least one criterion not met)<br>⇓<br>**Patient refused all post treatment lumbar punctures**<br>⇒ YES ⇒ ***Failure*** **(stop)**<br>⇓<br>All other cases ⇒ Failure	⇓<br>NO (no clinical signs or symptoms evoking a relapse at M18)<br>⇓<br>**WBC in CSF ≤20 at M12 or in absence of lumbar puncture at M12 and for both cases WBC in CSF ≤50 at M6 and no signs and symptoms at M18 evoking a relapse**:<br>⇒ YES ⇒ ***Success*** (Stop)<br>⇓<br>NO (at least one criterion not met)<br>⇓<br>**Patient refused all post treatment lumbar punctures**<br>⇒ YES ⇒ ***Failure*** (Stop)<br>⇓<br>All other cases ⇒ Failure

CSF=cerebrospinal fluid; g-HAT=human African trypanosomiasis due to *T. b. gambiense;* M=months; WBC=white blood cell.

Figure S7. Algorithm of classification to categorize patients as success or failure for the secondary effectiveness endpoint (12 months)

<table>
<tr><th>Derivation algorithm for
stage 1 and intermediate stage (Month 12)</th><th>Derivation algorithm for
stage 2 (Month 12)</th></tr>
<tr><td colspan="2"><u>Patient is dead</u> (any reason of death and any time between drug intake and M12)
⇒ YES ⇒ (definitive) Failure (stop)
⇓
NO (patient alive)
⇓
<u>Patient requires rescue medication for HAT at M12 or before</u>
⇒ YES ⇒ Failure (stop)
⇓
NO rescue medication so far
⇓
<u>Evidence of trypanosomes in any body fluid at M12 or between drug intake and M12 visit</u>
⇒ YES ⇒ (definitive) Failure (stop)
⇓
NO observed trypanosomes so far
⇓
<u>Patient Lost to follow-up at 12M </u>(no survival information at M12 and later)
⇒ YES ⇒ Failure (Stop)
⇓
NO (the patient is not lost to follow-up)</td></tr>
<tr><td>⇓
<u>Non-haemorrhagic lumbar puncture at M12 and WBC in CSF >20 cells</u>
⇒YES ⇒ Failure (stop)
⇓
NO (WBC in CSF at M12 ≤20 cells or no reliable count of WBC in CSF at M12)
⇓
<u>Non-haemorrhagic lumbar puncture at M12 and WBC in CSF at M12 ≤20 cells</u>
⇒ YES ⇒ Success (Stop)
⇓
NO (no reliable count of WBC in CSF at M12)
⇓
⇓
⇓
⇓</td><td>⇓
<u>Non-haemorrhagic lumbar puncture at M12 and WBC in CSF at M12 ≥ 50 cells</u>
⇒ YES ⇒ Failure (stop)
⇓
NO (WBC in CSF at M12 <50 cells or no reliable count of WBC in CSF at M12)
⇓
<u>Non-haemorrhagic lumbar puncture at M12 and WBC in CSF at M12 ≤20 cells</u>
⇒ YES ⇒ Success (Stop)
⇓
NO (no reliable count of WBC in CSF at M12)
⇓
<u>Non-haemorrhagic lumbar puncture at M12 with WBC in CSF <50 but >20 and increase of WBC in CSF with respect to M6 or sign and symptoms evoking a failure</u>
⇒ YES ⇒ Failure (stop)
⇓
NO (patient did not meet at least one of the previous criteria at M12)
⇓
<u>Non-haemorrhagic lumbar puncture at M12 with WBC in CSF<50 but >20 cells and decrease of WBC in CSF with respect to M6 and no signs and symptoms evoking a failure (success at M18 if status is available)</u>
⇒ YES ⇒ Success (stop)
⇓
NO (patient did not meet at least one of the previous criteria)</td></tr>
</table>

Derivation algorithm for stage 1 and intermediate stage (Month 12)	Derivation algorithm for stage 2 (Month 12)
⇓<br>**No lumbar puncture at M12 or no reliable count of WBC in CSF at M12 but reliable number of WBC in CSF reported later** (M18 or other additional visit)<br>⇒ YES ⇒ **WBC in CSF >20** ⇒ ***Failure* (Stop)**<br>⇒ YES ⇒ **WBC in CSF ≤20 and no signs or symptoms evoking a relapse** ⇒ ***Success* (Stop)**<br>⇓<br>NO (no later reliable count of WBC in CSF)<br>⇓	⇓<br>**No lumbar puncture at M12 or no reliable count of WBC in CSF at M12 but reliable number of WBC in CSF reported later** (M18 or other additional visit)<br>⇒ YES ⇒ **WBC in CSF >20** ⇒ ***Failure* (Stop)**<br>⇒ YES ⇒ **WBC in CSF ≤20** ⇒ ***Success* (Stop)**<br>⇓<br>NO (no later reliable count of WBC in CSF)<br>⇓
**No reliable WBC count in CSF at M12 and later and Failure at M18 for any reason**<br>⇒ YES ⇒ ***Failure*** at M12 **(Stop)**<br>⇓<br>NO<br>⇓	**No WBC count in CSF at M12 and later and Failure at M18 for any reason**<br>⇒ YES ⇒ ***Failure*** at M12 **(Stop)**<br>⇓<br>NO<br>⇓
⇓	**No reliable count of WBC in CSF at M18 and M12 but WBC counts at M6 available and no sign and symptoms evoking relapse at M12 or M18**<br>⇒ YES ⇒ **WBC in CSF at M6 ≤20 cells** ⇒ ***Success (stop)***<br>⇓<br>NO ⇒ **WBC in CSF at M6 >20 cells ⇒ *Failure* (Stop)**<br>⇓<br>NO (no lumbar puncture at M6, M12 and M18)<br>⇓
**Patient refused all post-treatment lumbar punctures but was met at M24 or later with no signs and symptoms evoking a relapse (normal activity)**<br>⇒ YES ⇒ ***Success*** at M12<br>⇓<br>NO (Patient not met at M24 or later)<br>⇓	**Patient refused all post-treatment lumbar punctures but was met at M24 or later with no signs and symptoms evoking a relapse (normal activity)**<br>**⇒ YES ⇒ *Success*** at M12<br>⇓<br>NO (Patient not met at M24 or later)<br>⇓
**Patient refused all post treatment lumbar punctures**<br>⇒ YES ⇒ ***Failure* (Stop)**	**Patient refused all post treatment lumbar punctures**<br>⇒ YES ⇒ ***Failure* (Stop)**

CSF=cerebrospinal fluid; g-HAT=human African trypanosomiasis due to *T. b. gambiense;* M=months; WBC=white blood cell.

Text S8. Selection of outpatients

Regarding the selection of outpatients, the following precautions were taken to ensure that outpatients would be capable of taking their daily dose soon after the main meal:

- Specific eligibility criteria for outpatients

As stated in the methods (subsection "Study design and participants"), specific, slightly more restrictive eligibility criteria were defined for outpatients, including proof that both outpatients and caregivers could understand treatment administration through a questionnaire, and that patients with neurological symptoms or medical/psychiatric contraindications for treatment at home could not be enrolled as outpatients.

- All outpatients had to be accompanied by a caregiver

The caregiver's role was to look after the patient during the treatment period and, more specifically, to ensure that fexinidazole was taken according to instructions. If several persons accompanied the patient, a choice had to be made by the patient (if possible) together with the Investigator to designate the most suitable person to fulfil the role.

- Body weight: patients had to weigh at least 20 kg to be enrolled in the study, since children and adolescents could be included. There was no minimal BMI set up for the outpatient cohort. But the capacity to ingest at least one solid meal per day was required to be able to enter the study.

Therefore, the recommendations from the WHO to have appropriate food intake and no history of psychiatric disorder were taken on board. But instead of requiring close medical supervision, we relied on giving careful instructions before treatment (see below) and involving a caregiver to ensure these instructions would be followed at home.

Regarding the monitoring of treatment adherence according to the instructions (including taking fexinidazole with a meal), this was carefully assessed before and after treatment:

- Questionnaire before treatment to assess feasibility (Table S9a).

A questionnaire was filled by the patients identified for treatment at home and their caregivers to check that the instructions for use were understood (8 questions in total). Patients had to answer questions such as "how many days in total do you have to take your treatment?", "do you have to take the same number of tablets every day?", or "do you have to take your treatment during or outside a meal?" If there was a doubt or a wrong answer, instructions were explained again by the staff. An instruction sheet was also distributed to the patients; they could refer to it during treatment.

- Interview and questionnaire after treatment to assess adherence (Table S9b)

The structured interview aimed at checking that the patients took their treatment as instructed (during a meal, for 10 days without interruption, etc.).

- Adherence to treatment was also monitored through PK endpoints (presence of fexinidazole and/or its main metabolites in the blood at end of treatment, which showed no difference between inpatients and outpatients, see figures S14 to S16) and the number of tablets returned at end of treatment.

Supported by these precautions, all 38 outpatients completed the full course of study treatment. Three patients were hospitalised due to an AE or at the investigator's discretion, which means that 35 outpatients completed the end-of-treatment interview; all of them complied with the dosing regimen, including taking fexinidazole during a meal. The pre- and post-treatment questionnaires have been added to the supplementary materials in Tables S8 and S9).

Table S9a. Pre-treatment questionnaire to check that instructions for use of fexinidazole were understood by outpatients (Dispensing visit, day 0)

With the support of the packaging, was the immediate answer from the patient or from the caregiver correct? YES/NO.
If the answer is not correct, explain again until the patient or the caregiver understands. Add in the comment section the difficulties faced.

Questions to be completed with the patient or the caregiver *(tick the appropriate box)*

**Q1** – During how many days in total do you have to take your treatment?<br>*Expected answer: 10 days*		
Was the immediate answer correct?	o YES<br>o NO	Comment:…………………………………………………
**Q2** – During the treatment period, do you have to take the same number of tablets every day? *Expected answer: No.*		
Was the immediate answer correct?	o YES<br>o NO	Comment:…………………………………………………
**Q3** – How many phases the treatment is composed of?<br>*Expected answer: 2 phases*		
Was the immediate answer correct?	o YES<br>o NO	Comment:…………………………………………………
**Q4** – How many tablets do you have to take on the first day of treatment?<br>*Expected answer for an adult: 3 tablets*<br>*Expected answer for a child: 2 tablets*		
Was the immediate answer correct?	o YES<br>o NO	Comment:…………………………………………………
**Q5** - How many tablets do you have to take on the 5th day of treatment?<br>*Expected answer for an adult: 2 tablets*<br>*Expected answer for a child: 1 tablet*		
Was the immediate answer correct?	o YES<br>o NO	Comment:…………………………………………………
**Q6** – Do you have to take your treatment during or outside a meal?<br>*Expected answer: During a meal.*		
Was the immediate answer correct?	o YES<br>o NO	Comment:…………………………………………………
**Q7** – Each day, do you have to take all the tablets at the same time (swallowing them one after the other)?<br>*Expected answer: YES*		
Was the immediate answer correct?	o YES<br>o NO	Comment:…………………………………………………
**Q8** – With the support of the packaging, explain briefly how you are going to take your treatment.<br>*Expected answer for the adults: 2 phases. Day 1 to 4 = 3 tablets. Day 5 to 10 = 2 tablets. Tablets to be taken all at once during a meal.*<br>*Expected answer for a child: 2 phases. Day 1 to 4 = 2 tablets. Day 5 to 10 = 1 tablet. Tablets to be taken all at once during a meal.*		
Was the immediate answer correct?	o YES<br>o NO	Comment:…………………………………………………

Conclusion:

- Person who answered the questionnaire:
  - o Patient
  - o Caregiver
  - o Both the patient and the caregiver
- Is patient/caregiver autonomous to take the treatment at home?
  - o YES
  - o NO

If YES, which factor defines the autonomy for taking the treatment at home:
- o the understanding of the patient alone
- o with the help of the caregiver

Table S8b. Post-treatment questionnaire to assess outpatients' adherence to treatment and packaging acceptability (End of Treatment visit, day 11)

Patient and caregiver must present the treatment packaging they received at study inclusion. Add in the comment section the difficulties faced or the information deemed relevant.

Question to be completed by the investigator *(tick the appropriate box)*

**Q1** – Was the calendar completed?	o YES<br>o NO<br>o PARTIALLY	Comment:………………………………………………

Questions to be completed with the patient or the caregiver *(tick the appropriate box)*

**Q2** – Did you find the explanation provided before starting the treatment helpful?	o YES<br>o NO	Comment:………………………………………………
**Q3** – Did you have to request help to follow the treatment?	o YES<br>o NO	Comment:………………………………………………
**Q4** – Did you find the instruction sheet provided with the medication helpful / did it help you to remember the important information?	o YES<br>o NO	Comment:………………………………………………
**Q5** – How did you take your treatment?<br>*Expected answer for the adults: 2 phases. Day 1 to 4 = 3 tablets. Day 5 to 10 = 2 tablets. Tablets to be taken all at once during a meal.*<br>*Expected answer for the children: 2 phases. Day 1 to 4 = 2 tablets. Day 5 to 10 = 1 tablet. Tablets to be taken all at once during a meal*		
Was the immediate answer correct?	o YES<br>o NO	Comment:………………………………………………<br>Have the 4 key messages been understood?<br>1-The treatment period is composed of 2 phases, each with a different number of tablets to be taken<br>□ YES □ NO<br>2-The treatment is to be taken for 10 days without interruption<br>□ YES □ NO<br>3-The tablets have to be taken all at once every day?<br>□ YES □ NO<br>4-The treatment is to be taken during a meal<br>□ YES □ NO

Text S10. Baseline clinical characteristics according to g-HAT stage and change in prevalence over time (Table S11a and Table S11b)

No major differences between g-HAT stage subgroups were observed for the unspecific most frequent symptoms (Table S11a). However, several signs/symptoms reported by the patients, including g-HAT-characteristic neuropsychiatric manifestations, showed a clearly higher prevalence in patients with stage 2 g-HAT: drowsiness (78% of the 104 patients with stage 2 g-HAT versus 21% of the 70 patients with stage 1/intermediate g-HAT), pruritus (62% versus 20%), asthenia (58% versus 30%), tremor* (27% versus 1%), and behavioural disturbances* (21% versus 4%). Consistent with the higher prevalence of pruritus, skin abnormalities (mainly scratch marks) were also more frequent in patients with stage 2 g-HAT (46%) than in patients with stage 1/intermediate g-HAT (17%) (Table S11b).

A total of 106 of 174 patients (61%) had a fully normal neuropsychiatric examination, with a lower frequency in patients with stage 2 g-HAT (44 of 104 patients, 42%) than in patients with stage 1/intermediate g-HAT (62 of the 70 patients, 89%). The largest differences between stages were observed for the prevalence of tremor* (30% of patients with stage 2 g-HAT versus 3% of patients with stage 1/intermediate g-HAT), rapid alternating movements (24% versus 1%), abnormal verbal flow (21% versus 4%), involuntary movements (16% versus 0%), primitive palm-chin reflex (15% versus 0%), and behavioural disturbances* (14% versus 4%) (Table S11b).

The prevalence of clinical signs and symptoms of g-HAT as well as abnormalities observed during physical and neurological examination decreased significantly after completion of fexinidazole treatment. Apart from headaches and fever, the largest decrease in prevalence after 10 days of treatment was observed for drowsiness (from 55% to 6%), mainly due to the decrease in prevalence in stage 2 patients (from 78% to 10% at the end of treatment). The prevalence of nocturnal sleep disturbances (insomnia) also decreased after 10 days (from 24% to 12%) (Table S11a). After 3 months, a further improvement was observed, with almost all patients having normal examination apart from the persistence of swollen cervical lymph nodes (29% of patients) and abnormal skin examination in patients with stage 2 g-HAT (14%, along with pruritus, still present in 12%). Abnormalities at 18 months tended to be very rare and unspecific (Table S11a and Table S11b).

* Tremor and behavioural disturbances were signs and symptoms of HAT potentially reported by the patient, but also potential findings of the neurological examination performed by the investigator. Data about HAT signs/symptoms and neurological examination were reported in two separate sections of the case report form. This explains the slight discrepancies in the prevalence of tremor and behavioural disturbances between the two evaluations.

Table S11a . Prevalence of clinical signs and symptoms of g-HAT over time, by cohort, g-HAT stage, and overall in the modified intent-to-treat population

	Inpatients N=136	Outpatients N=38	Stage 1 or intermediate N=70	Stage 2 N=104	Total N=174
**Headaches**					
Baseline	97 (71%)	27 (71%)	53 (76%)	71 (68%)	124 (71%)
EoT	5 (4%)	7 (19%)	6 (9%)	6 (6%)	12 (7%)
Month 3	4 (3%)	2 (5%)	6 (9%)	0	6 (4%)
Month 18	1 (1%)	1 (3%)	2 (3%)	0	2 (1%)
**Fever**					
Baseline	87 (64%)	21 (55%)	46 (66%)	62 (60%)	108 (62%)
EoT	3 (2%)	3 (8%)	5 (7%)	1 (1%)	6 (4%)
Month 3	5 (4%)	2 (5%)	5 (7%)	2 (2%)	7 (4%)
Month 18	4 (3%)	1 (3%)	3 (5%)	2 (2%)	5 (3%)
**Drowsiness**					
Baseline	74 (54%)	22 (58%)	15 (21%)	81 (78%)	96 (55%)
EoT	8 (6%)	2 (5%)	0	10 (10%)	10 (6%)
Month 3	1 (1%)	0	0	1 (1%)	1 (1%)
Month 18	0	0	0	0	0
**Asthenia**					
Baseline	67 (49%)	14 (37%)	21 (30%)	60 (58%)	81 (47%)
EoT	10 (8%)	7 (19%)	5 (7%)	12 (12%)	17 (10%)
Month 3	4 (3%)	0	1 (1%)	3 (3%)	4 (2%)
Month 18	0	1 (3%)	1 (2%)	0	1 (1%)
**Pruritus**					
Baseline	65 (48%)	13 (34%)	14 (20%)	64 (62%)	78 (45%)
EoT	24 (18%)	5 (14%)	2 (3%)	27 (27%)	29 (17%)
Month 3	10 (8%)	2 (5%)	0	12 (12%)	12 (7%)
Month 18	0	0	0	0	0
**Thinning/Weight Loss**					
Baseline	64 (47%)	12 (32%)	26 (37%)	50 (48%)	76 (44%)
EoT	18 (14%)	5 (14%)	11 (16%)	12 (12%)	23 (14%)
Month 3	10 (8%)	0	3 (4%)	7 (7%)	10 (6%)
Month 18	4 (3%)	0	0	4 (4%)	4 (2%)
**Insomnia**					
Baseline	37 (27%)	5 (13%)	17 (24%)	25 (24%)	42 (24%)
EoT	12 (9%)	8 (22%)	8 (12%)	12 (12%)	20 (12%)
Month 3	1 (1%)	0	0	1 (1%)	1 (1%)
Month 18	0	0	0	0	0
**Amenorrhoea**[a]	N=51	N=9	N=25	N=35	N=60
Baseline	13 (25%)	1 (11%)	5 (20%)	9 (26%)	14 (23%)
EoT	11 (21%)	1 (11%)	5 (19%)	7 (19%)	12 (19%)
Month 3	1 (2%)	1 (10%)	2 (8%)	0	2 (3%)
Month 18	4 (8%)	1 (8%)	3 (10%)	2 (6%)	5 (8%)

	Inpatients N=136	Outpatients N=38	Stage 1 or intermediate N=70	Stage 2 N=104	Total N=174
**Anorexia**					
Baseline	25 (18%)	7 (18%)	13 (19%)	19 (18%)	32 (18%)
EoT	7 (5%)	6 (16%)	6 (9%)	7 (7%)	13 (8%)
Month 3	2 (2%)	0	1 (1%)	1 (1%)	2 (1%)
Month 18	1 (1%)	0	0	1 (1%)	1 (1%)
**Tremor**					
Baseline	22 (16%)	7 (18%)	1 (1%)	28 (27%)	29 (17%)
EoT	9 (7%)	6 (16%)	1 (1%)	14 (14%)	15 (9%)
Month 3	0	0	0	0	0
Month 18	0	0	0	0	0
**Sexual Impotence**[b]	N=42	N=17	N=23	N=36	N=59
Baseline	7 (17%)	2 (12%)	3 (13%)	6 (17%)	9 (15%)
EoT	5 (12%)	2 (11%)	2 (9%)	5 (14%)	7 (12%)
Month 3	0	1 (6%)	1 (4%)	0	1 (2%)
Month 18	0	1 (7%)	1 (5%)	0	1 (2%)
**Behavioural Disturbances**					
Baseline	18 (13%)	7 (18%)	3 (4%)	22 (21%)	25 (14%)
EoT	7 (5%)	2 (5%)	0	9 (9%)	9 (5%)
Month 3	4 (3%)	0	0	4 (4%)	4 (2%)
Month 18	1 (1%)	0	0	1 (1%)	1 (1%)
**Speech Disturbances**					
Baseline	11 (8%)	3 (8%)	2 (3%)	12 (12%)	14 (8%)
EoT	0	1 (3%)	0	1 (1%)	1 (1%)
Month 3	1 (1%)	0	0	1 (1%)	1 (1%)
Month 18	0	0	0	0	0
**Gait Disturbances**					
Baseline	10 (7%)	3 (8%)	0	13 (13%)	13 (7%)
EoT	5 (4%)	0	0	5 (5)	5 (3%)
Month 3	1 (1%)	0	0	1 (1)	1 (1%)
Month 18	0	0	0	0	0
**Nausea**					
Baseline	9 (7%)	3 (8%)	4 (6%)	8 (8%)	12 (7%)
EoT	4 (3%)	3 (8%)	4 (6%)	3 (3%)	7 (4%)
Month 3	0	0	0	0	0
Month 18	3 (2%)	0	1 (2%)	2 (2%)	3 (2%)
**Diarrhoea**					
Baseline	4 (3%)	2 (5%)	1 (1%)	5 (5%)	6 (3%)
EoT	0	1 (3%)	1 (1%)	0	1 (1%)
Month 3	0	0	0	0	0
Month 18	0	0	0	0	0

	Inpatients N=136	Outpatients N=38	Stage 1 or intermediate N=70	Stage 2 N=104	Total N=174
**Convulsions**					
Baseline	2 (2%)	0	1 (1%)	1 (1%)	2 (1%)
EoT	0	0	0	0	0
Month 3	0	0	0	0	0
Month 18	0	0	0	0	0
**Other**					
Baseline	25 (18%)	12 (32%)	13 (19%)	24 (23%)	37 (21%)
EoT	5 (4%)	2 (5%)	2 (3%)	5 (5%)	7 (4%)
Month 3	1 (1%)	0	0	1 (1%)	1 (1%)
Month 18	0	0	0	0	0

a Prevalence calculated based on the total number of women older than 12 years and who were not pregnant/not menopausal at screening in the mITT population (n=60 at baseline).

b Prevalence calculated based on the total number of men older than 12 years in the mITT population (n=59 at baseline).

Data are presented as the number of patients (percentage of patients) presenting the sign or symptom.

The sample sizes that are presented (N=) are those at baseline. At baseline, there were no missing data. At EoT, data were missing for 5 patients (4 inpatients and 1 outpatient; 2 patients with stage 1/intermediate g-HAT and 3 patients with stage 2 g-HAT), except for amenorrhoea (data missing for 3 patients) and sexual impotence (data missing for 2 patients). At Month 3, data were missing for 3 patients (3 inpatients and 0 outpatient; 1 patient with stage 1/intermediate g-HAT and 2 patients with stage 2 g-HAT), except for amenorrhoea (data missing for 2 patients) and sexual impotence (data missing for 1 patient). At Month 18, data were missing for 11 patients (8 inpatients and 3 outpatients; 4 patients with stage 1/intermediate g-HAT and 7 patients with stage 2 g-HAT), except for amenorrhoea (data missing for 1 patient) and sexual impotence (data missing for 10 patients).

The modified intent-to-treat population included all patients who took at least one tablet of fexinidazole.

EoT=End of Treatment; g-HAT=human African trypanosomiasis due to *T. b.·gambiense*.

Table S11b.·Physical and neurological abnormalities present in at least 10% of patients in any subgroup at baseline, and change in prevalence over time by cohort, g-HAT stage, and overall in the modified intent-to-treat population

	Inpatients N=136	Outpatients N=38	Stage 1 or intermediate N=70	Stage 2 N=104	Total N=174
**Swollen cervical lymph nodes**					
Baseline	74 (54%)	20 (53%)	38 (54%)	56 (54%)	94 (54%)
EoT	63 (48%)	11 (30%)	31 (46%)	43 (43%)	74 (44%)
Month 3	40 (30%)	9 (24%)	18 (26%)	31 (30%)	49 (29%)
Month 18	3 (2%)	0	2 (3%)	1 (1%)	3 (2%)
**Abnormal abdominal palpation and percussion**[a]					
Baseline	14 (10%)	3 (8%)	8 (11%)	9 (9%)	17 (10%)
EoT	3 (2%)	0	2 (3%)	1 (1%)	3 (2%)
Month 3	4 (3%)	0	1 (1%)	3 (3%)	4 (2%)
Month 18	3 (2%)	1 (3%)	1 (2%)	3 (3%)	4 (2%)
**Skin abnormalities**[b]					
Baseline	50 (37%)	10 (26%)	12 (17%)	48 (46%)	60 (34%)
EoT	40 (30%)	7 (19%)	9 (13%)	38 (38%)	47 (28%)
Month 3	11 (8%)	5 (13%)	2 (3%)	14 (14%)	16 (9%)
Month 18	2 (2%)	0	0	2 (2%)	2 (1%)
**Abnormal verbal flow**					
Baseline	21 (15%)	4 (11%)	3 (4%)	22 (21%)	25 (14%)
EoT	5 (4%)	2 (5%)	1 (1%)	6 (6%)	7 (4%)
Month 3	3 (2%)	0	0	3 (3%)	3 (2%)
Month 18	0	0	0	0	0
**Behavioural disturbances**					
Baseline	13 (10%)	5 (13%)	3 (4%)	15 (14%)	18 (10%)
EoT	7 (5%)	2 (5%)	0	9 (9%)	9 (5%)
Month 3	5 (4%)	0	0	5 (5%)	5 (3%)
Month 18	1 (2%)	0	0	1 (1%)	1 (1%)
**Walking difficulty**					
Baseline	10 (7%)	2 (5%)	0	12 (12%)	12 (7%)
EoT	4 (3%)	0	0	4 (4%)	4 (2%)
Month 3	2 (2%)	0	0	2 (2%)	2 (1%)
Month 18	0	0	0	0	0
**Presence of involuntary movements**					
Baseline	12 (9%)	5 (13%)	0	17 (16%)	17 (10%)
EoT	9 (7%)	5 (14%)	1 (1%)	13 (13%)	14 (8%)
Month 3	0	0	0	0	0
Month 18	0	0	0	0	0
**Abnormal rapid alternating movements**					
Baseline	24 (18%)	2 (5%)	1 (1%)	25 (24%)	26 (15%)
EoT	12 (9%)	2 (5%)	0	14 (14%)	14 (8%)
Month 3	3 (2%)	0	0	3 (3%)	3 (2%)

	Inpatients N=136	Outpatients N=38	Stage 1 or intermediate N=70	Stage 2 N=104	Total N=174
Month 18	0	0	0	0	0
**Tremor**					
Baseline	27 (20%)	6 (16%)	2 (3%)	31 (30%)	33 (19%)
EoT	11 (8%)	7 (19%)	1 (1%)	17 (17%)	18 (11%)
Month 3	0	1 (3%)	1 (1%)	0	1 (1%)
Month 18	0	0	0	0	0
**Presence of primitive palm-chin reflex**					
Baseline	10 (7%)	6 (16%)	0	16 (15%)	16 (9%)
EoT	8 (6%)	5 (14%)	0	13 (13%)	13 (8%)
Month 3	0	0	0	0	0
Month 18	2 (2%)	0	0	2 (2%)	2 (1%)

a Abnormal abdominal palpation/percussion was due to the presence of splenomegaly and/or tenderness. At baseline, of the 17 patients with abnormalities, 9 had tenderness, 7 had splenomegaly, and 1 had tenderness/splenomegaly. Abnormal abdominal palpation/percussion present at baseline persisted after EoT in 2 patients (one who had splenomegaly until 12 months, and one who had tenderness until EoT and splenomegaly until EoH).

b Skin abnormalities were due to the presence of scratch marks and/or scabies. At baseline, of the 60 patients with skin abnormalities, 43 had scratch marks, 2 had scratch marks/scabies and 15 had other skin abnormalities.

Data are presented as the number of patients (percentage of patients) presenting the abnormality.

The sample sizes that are presented (N=) are those at baseline. At baseline, there were no missing data. At EoT, data were missing for 5 patients (4 inpatients and 1 outpatient; 2 patients with stage 1/intermediate g-HAT and 3 patients with stage 2 g-HAT). At Month 3, data were missing for 3 patients (3 inpatients and 0 outpatient; 1 patient with stage 1/intermediate g-HAT and 2 patients with stage 2 g-HAT). At Month 18, data were missing for 11 patients (8 inpatients and 3 outpatients; 4 patients with stage 1/intermediate g-HAT and 7 patients with stage 2 g-HAT).

The modified intent-to-treat population included all patients who took at least one tablet of fexinidazole.

EoH=End of Hospitalisation; EoT=End of Treatment; g-HAT=human African trypanosomiasis due to *T. b. gambiense*.

Table S9. Relationship between failure rate at 18 months and potential predictors: results of the Poisson regression model and generalized additive models (spline)

	**Baseline CSF WBC**	**Adherence[a]**	**Baseline symptom score[b]**
Failure rate at 18 months			
Adjusted[c] risk ratio	1.00	Not performed	1.00
95% CI	[1.00 ; 1.00]		[0.99; 1.00]
p-value	0.92		0.52

a Adherence, calculated as the number of tablets left over at the end of treatment, was not included in the model because only one patient did not complete the full course of treatment.
b The symptom score was calculated as the sum of weighted symptoms.
c From a multivariate Poisson model including baseline CSF WBC and baseline symptom score and adjusted on age, sex, BMI, and patients' cohort (inpatients / outpatients).

Generalized additive models including baseline CSF WBC and baseline symptom score, and adjusted for age, sex, BMI, and patient cohort (inpatients / outpatients), were performed to take into account non-linear data dependency structure. We used degrees of freedom = 2 and 3, corresponding to a smoothing spline with the complexity of a quadratic polynomial and cubic polynomial respectively. The results show that there were no significant effects of the baseline CSF WBC (p=0.58) and baseline symptom score (p= 0.75) for linear terms on failure rate at 18 months.
CSF=cerebrospinal fluid; g-HAT=human African trypanosomiasis due to *T. b. gambiense;* WBC=white blood cell.

Table S10. All treatment-emergent adverse events by system organ class and preferred term in the modified intent-to-treat population

Primary SOC<br>PT	Inpatients (N=136)	Outpatients (N=38)	Stage 1 or intermediate g-HAT (N=70)	Stage 2 g-HAT (N=104)	Total (N=174)
Any AE	84 (62%) [273]	26 (68%) [75]	49 (70%) [160]	61 (59%) [188]	110 (63%) [348]
Gastrointestinal disorders	53 (39%) [92]	13 (34%) [22]	33 (47%) [62]	33 (32%) [52]	66 (38%) [114]
Vomiting	35 (26%) [41]	6 (16%) [6]	21 (30%) [23]	20 (19%) [24]	41 (24%) [47]
Nausea	20 (15%) [24]	7 (18%) [7]	15 (21%) [17]	12 (12%) [14]	27 (16%) [31]
Abdominal pain	6 (4%) [6]	2 (5%) [2]	4 (6%) [4]	4 (4%) [4]	8 (5%) [8]
Dyspepsia	6 (4%) [6]	2 (5%) [2]	6 (9%) [6]	2 (2%) [2]	8 (5%) [8]
Abdominal pain upper	5 (4%) [6]	2 (5%) [2]	5 (7%) [6]	2 (2%) [2]	7 (4%) [8]
Salivary hypersecretion	4 (3%) [4]	2 (5%) [2]	2 (3%) [2]	4 (4%) [4]	6 (3%) [6]
Diarrhoea	1 (1%) [1]	1 (3%) [1]	1 (1%) [1]	1 (1%) [1]	2 (1%) [2]
Dysphagia	1 (1%) [2]	0	1 (1%) [2]	0	1 (1%) [2]
Constipation	1 (1%) [1]	0	0	1 (1%) [1]	1 (1%) [1]
Gastritis	1 (1%) [1]	0	1 (1%) [1]	0	1 (1%) [1]
Nervous system disorders	36 (26%) [47]	12 (32%) [15]	24 (34%) [27]	24 (23%) [35]	48 (28%) [62]
Headache	24 (18%) [30]	8 (21%) [8]	15 (21%) [16]	17 (16%) [22]	32 (18%) [38]
Dizziness	10 (7%) [11]	2 (5%) [2]	7 (10%) [8]	5 (5%) [5]	12 (7%) [13]
Tremor	0	4 (11%) [4]	1 (1%) [1]	3 (3%) [3]	4 (2%) [4]
Epilepsy	2 (1%) [2]	0	1 (1%) [1]	1 (1%) [1]	2 (1%) [2]
Amnesia	1 (1%) [1]	0	0	1 (1%) [1]	1 (1%) [1]
Paraesthesia	0	1 (3%) [1]	1 (1%) [1]	0	1 (1%) [1]
Psychomotor hyperactivity	1 (1%) [1]	0	0	1 (1%) [1]	1 (1%) [1]
Psychomotor skills impaired	1 (1%) [1]	0	0	1 (1%) [1]	1 (1%) [1]
Seizure	1 (1%) [1]	0	0	1 (1%) [1]	1 (1%) [1]
General disorders and administration site conditions	34 (25%) [43]	9 (24%) [9]	18 (26%) [21]	25 (24%) [31]	43 (25%) [52]
Asthenia	22 (16%) [24]	1 (3%) [1]	12 (17%) [13]	11 (11%) [12]	23 (13%) [25]
Pyrexia	16 (12%) [16]	3 (8%) [3]	3 (4%) [3]	16 (15%) [16]	19 (11%) [19]
Feeling hot	2 (1%) [2]	2 (5%) [2]	3 (4%) [3]	1 (1%) [1]	4 (2%) [4]
Chills	1 (1%) [1]	0	0	1 (1%) [1]	1 (1%) [1]
Feeling cold	0	1 (3%) [1]	1 (1%) [1]	0	1 (1%) [1]
Influenza-like illness	0	1 (3%) [1]	0	1 (1%) [1]	1 (1%) [1]
Pain	0	1 (3%) [1]	1 (1%) [1]	0	1 (1%) [1]

Primary SOC<br>PT	Inpatients (N=136)	Outpatients (N=38)	Stage 1 or intermediate g-HAT (N=70)	Stage 2 g-HAT (N=104)	Total (N=174)
Psychiatric disorders	21 (15%) [31]	11 (29%) [15]	13 (19%) [20]	19 (18%) [26]	32 (18%) [46]
Insomnia	12 (9%) [12]	7 (18%) [7]	8 (11%) [8]	11 (11%) [11]	19 (11%) [19]
Anxiety	9 (7%) [11]	3 (8%) [3]	6 (9%) [8]	6 (6%) [6]	12 (7%) [14]
Agitation	1 (1%) [2]	1 (3%) [1]	1 (1%) [2]	1 (1%) [1]	2 (1%) [3]
Abnormal behaviour	1 (1%) [1]	1 (3%) [1]	0	2 (2%) [2]	2 (1%) [2]
Confusional state	1 (1%) [1]	1 (3%) [1]	1 (1%) [1]	1 (1%) [1]	2 (1%) [2]
Hallucination	1 (1%) [1]	1 (3%) [1]	1 (1%) [1]	1 (1%) [1]	2 (1%) [2]
Mood swings	2 (1%) [2]	0	0	2 (2%) [2]	2 (1%) [2]
Hallucination, visual	0	1 (3%) [1]	0	1 (1%) [1]	1 (1%) [1]
Psychotic disorder due to a general medical condition	1 (1%) [1]	0	0	1 (1%) [1]	1 (1%) [1]
Metabolism and nutrition disorders	13 (10%) [13]	3 (8%) [3]	7 (10%) [7]	9 (9%) [9]	16 (9%) [16]
Decreased appetite	10 (7%) [10]	3 (8%) [3]	4 (6%) [4]	9 (9%) [9]	13 (7%) [13]
Dehydration	1 (1%) [1]	0	1 (1%) [1]	0	1 (1%) [1]
Diabetes mellitus	1 (1%) [1]	0	1 (1%) [1]	0	1 (1%) [1]
Starvation	1 (1%) [1]	0	1 (1%) [1]	0	1 (1%) [1]
Infections and infestations	9 (7%) [9]	3 (8%) [3]	4 (6%) [4]	8 (8%) [8]	12 (7%) [12]
Malaria	5 (4%) [5]	1 (3%) [1]	2 (3%) [2]	4 (4%) [4]	6 (3%) [6]
Appendicitis	0	1 (3%) [1]	0	1 (1%) [1]	1 (1%) [1]
Cholera	0	1 (3%) [1]	1 (1%) [1]	0	1 (1%) [1]
Conjunctivitis	1 (1%) [1]	0	0	1 (1%) [1]	1 (1%) [1]
Furuncle	1 (1%) [1]	0	0	1 (1%) [1]	1 (1%) [1]
Tuberculous pleurisy	1 (1%) [1]	0	1 (1%) [1]	0	1 (1%) [1]
Urinary tract infection	1 (1%) [1]	0	0	1 (1%) [1]	1 (1%) [1]
Musculoskeletal and connective tissue disorders	10 (7%) [11]	1 (3%) [1]	6 (9%) [6]	5 (5%) [6]	11 (6%) [12]
Back pain	7 (5%) [8]	1 (3%) [1]	5 (7%) [5]	3 (3%) [4]	8 (5%) [9]
Neck pain	2 (1%) [2]	0	1 (1%) [1]	1 (1%) [1]	2 (1%) [2]
Muscle spasms	1 (1%) [1]	0	0	1 (1%) [1]	1 (1%) [1]
Respiratory, thoracic and mediastinal disorders	7 (5%) [8]	0	0	7 (7%) [8]	7 (4%) [8]
Cough	3 (2%) [3]	0	0	3 (3%) [3]	3 (2%) [3]
Dry throat	1 (1%) [1]	0	0	1 (1%) [1]	1 (1%) [1]
Dysphonia	1 (1%) [1]	0	0	1 (1%) [1]	1 (1%) [1]
Dyspnoea exertional	1 (1%) [1]	0	0	1 (1%) [1]	1 (1%) [1]
Epistaxis	1 (1%) [1]	0	0	1 (1%) [1]	1 (1%) [1]
Rhinorrhoea	1 (1%) [1]	0	0	1 (1%) [1]	1 (1%) [1]
Blood and lymphatic system disorders	4 (3%) [5]	0	3 (4%) [4]	1 (1%) [1]	4 (2%) [5]
Anaemia	4 (3%) [4]	0	3 (4%) [3]	1 (1%) [1]	4 (2%) [4]
Lymphadenopathy	1 (1%) [1]	0	1 (1%) [1]	0	1 (1%) [1]
Vascular disorders	1 (1%) [2]	2 (5%) [2]	1 (1%) [1]	2 (2%) [3]	3 (2%) [4]
Hot flush	1 (1%) [2]	2 (5%) [2]	1 (1%) [1]	2 (2%) [3]	3 (2%) [4]

Primary SOC<br>PT	Inpatients (N=136)	Outpatients (N=38)	Stage 1 or intermediate g-HAT (N=70)	Stage 2 g-HAT (N=104)	Total (N=174)
Investigations	3 (2%) [3]	0	1 (1%) [1]	2 (2%) [2]	3 (2%) [3]
Weight decreased	2 (1%) [2]	0	0	2 (2%) [2]	2 (1%) [2]
Blood potassium increased	1 (1%) [1]	0	1 (1%) [1]	0	1 (1%) [1]
Renal and urinary disorders	1 (1%) [1]	1 (3%) [1]	2 (3%) [2]	0	2 (1%) [2]
Dysuria	1 (1%) [1]	1 (3%) [1]	2 (3%) [2]	0	2 (1%) [2]
Skin and subcutaneous tissue disorders	2 (1%) [2]	1 (3%) [2]	1 (1%) [1]	2 (2%) [3]	3 (2%) [4]
Hyperhidrosis	0	1 (3%) [1]	0	1 (1%) [1]	1 (1%) [1]
Papule	1 (1%) [1]	0	0	1 (1%) [1]	1 (1%) [1]
Pruritus	0	1 (3%) [1]	0	1 (1%) [1]	1 (1%) [1]
Skin disorder	1 (1%) [1]	0	1 (1%) [1]	0	1 (1%) [1]
Cardiac disorders	2 (1%) [2]	0	1 (1%) [1]	1 (1%) [1]	2 (1%) [2]
Myocardial ischaemia	1 (1%) [1]	0	0	1 (1%) [1]	1 (1%) [1]
Palpitations	1 (1%) [1]	0	1 (1%) [1]	0	1 (1%) [1]
Pregnancy, puerperium and perinatal conditions	2 (1%) [2]	0	1 (1%) [1]	1 (1%) [1]	2 (1%) [2]
Stillbirth	1 (1%) [1]	0	0	1 (1%) [1]	1 (1%) [1]
Transverse presentation	1 (1%) [1]	0	1 (1%) [1]	0	1 (1%) [1]
Injury, poisoning and procedural complications	1 (1%) [2]	0	0	1 (1%) [2]	1 (1%) [2]
Uterine rupture	1 (1%) [1]	0	0	1 (1%) [1]	1 (1%) [1]
Wound	1 (1%) [1]	0	0	1 (1%) [1]	1 (1%) [1]
Reproductive system and breast disorders	0	1 (3%) [1]	1 (1%) [1]	0	1 (1%) [1]
Vaginal discharge	0	1 (3%) [1]	1 (1%) [1]	0	1 (1%) [1]
Ear and labyrinth disorders	0	1 (3%) [1]	1 (1%) [1]	0	1 (1%) [1]
Tinnitus	0	1 (3%) [1]	1 (1%) [1]	0	1 (1%) [1]

Data are presented as the number of patients (percentage of patients) [number of treatment-emergent events].
Dictionary used MedDRA version 19.1.
The modified intent-to-treat population included all patients who took at least one tablet of fexinidazole.
AE=adverse event; g-HAT=human African trypanosomiasis due to *T. b. gambiense*; MedDRA=Medical Dictionary for Regulatory Activities; PT=preferred term; SOC=system organ class.

Figure S11. Distribution of fexinidazole concentration by visit for in/out-patients in the PK population

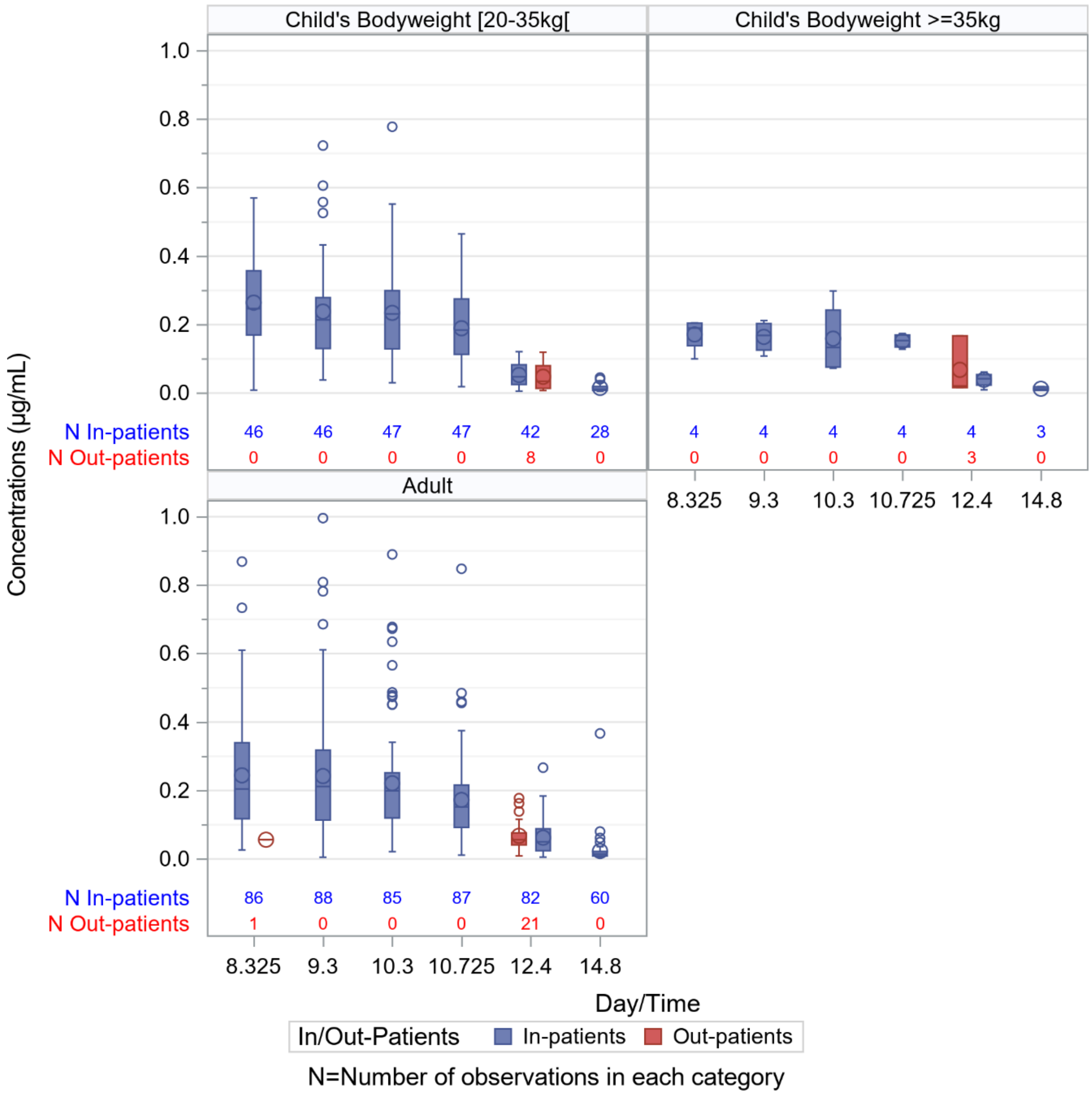

Each box displays the median (line within the box), the mean (big blue circle/red cross within the box) and the 25th and 75th percentiles (extremities of the box). The whiskers display the minimum and maximum values (excluding outliers). Outliers are defined as any value below or above 1.5 * interquartile range (from 25th to 75th percentiles) and are displayed with small blue circles/red crosses.
D=day; h=hour; PK=pharmacokinetic; T=time.

Figure S12. Distribution of M1 concentration by visit for in/out-patients in the PK population

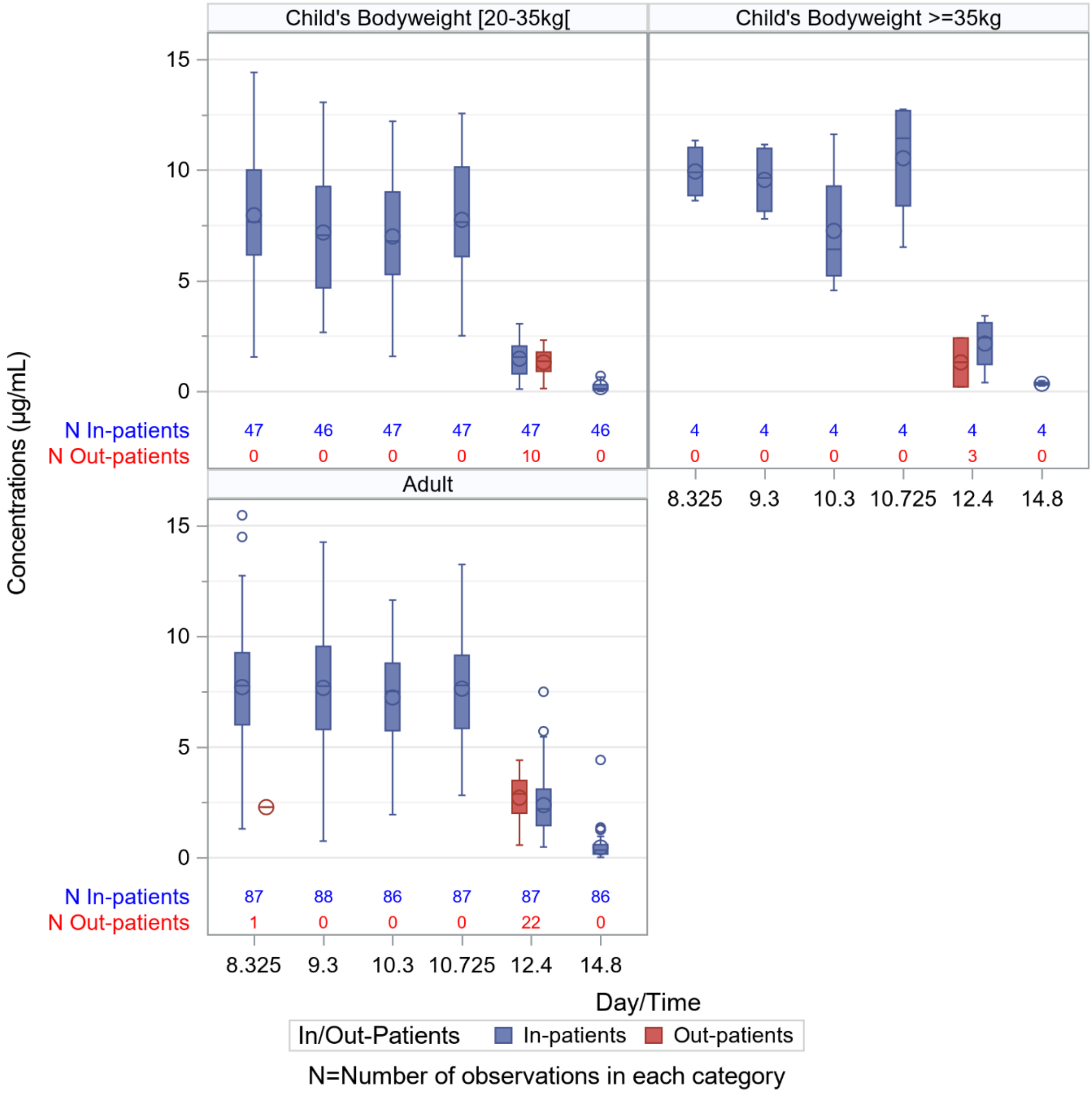

Each box displays the median (line within the box), the mean (big blue circle/red cross within the box) and the 25th and 75th percentiles (extremities of the box). The whiskers display the minimum and maximum values (excluding outliers). Outliers are defined as any value below or above 1.5 * interquartile range and are displayed with small blue circles/red crosses.

D=day; h=hour; M1= fexinidazole sulfoxide; PK=pharmacokinetic; T=time.

Figure S13. Distribution of M2 concentration by visit for in/out-patients in the PK population

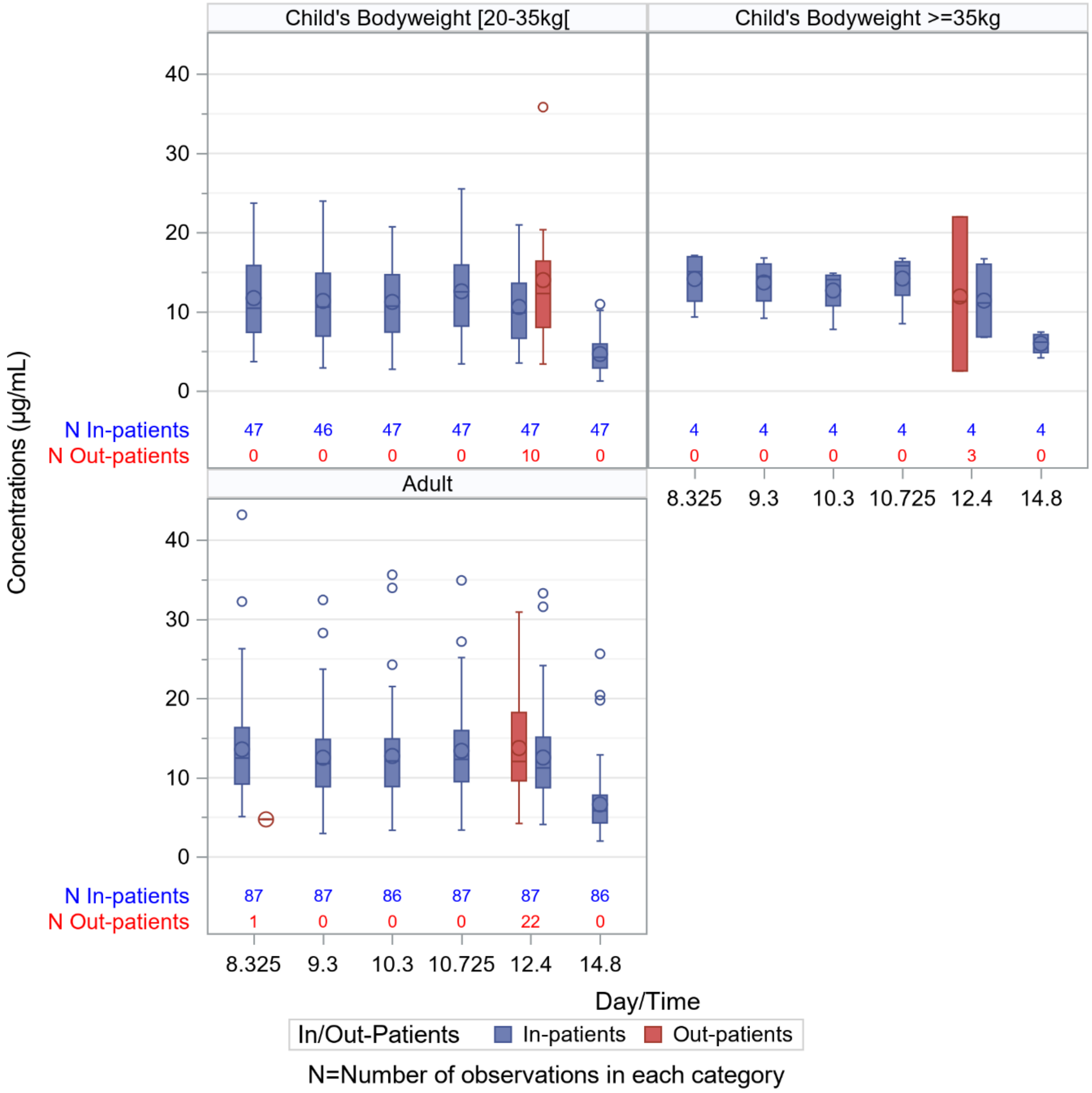

Each box displays the median (line within the box), the mean (big blue circle/red cross within the box) and the 25th and 75th percentiles (extremities of the box). The whiskers display the minimum and maximum values (excluding outliers). Outliers are defined as any value below or above 1.5 * interquartile range and are displayed with small blue circles/red crosses.
D=day; h=hour; M2=fexinidazole sulfone; PK=pharmacokinetic; T=time.

Figure S14. Concentrations of fexinidazole, M1, and M2: Comparison of patients with g-HAT relapse to the median, 5th and 95th percentiles of all patients in the PK population

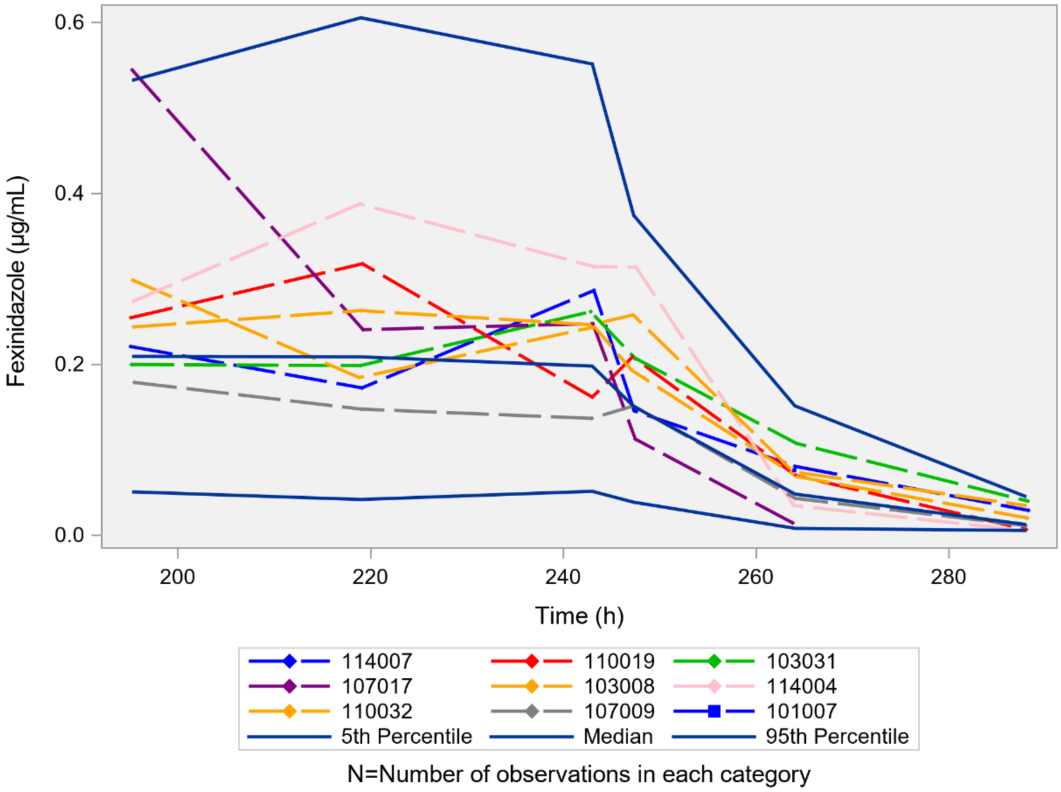

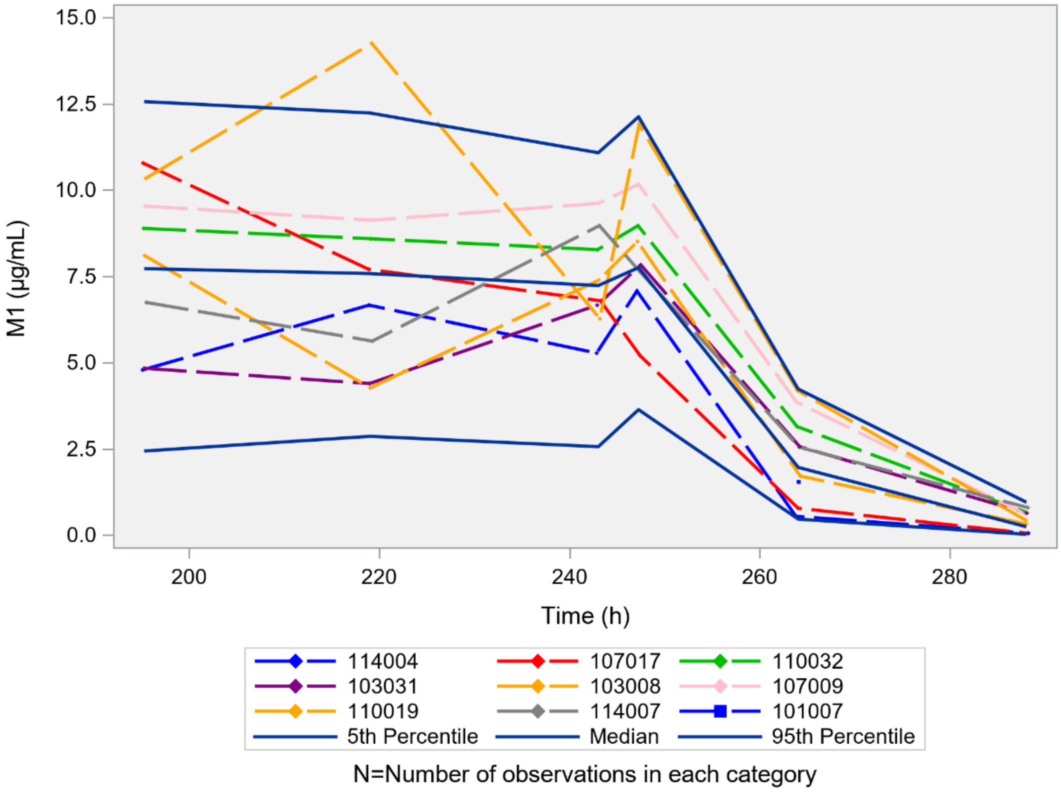

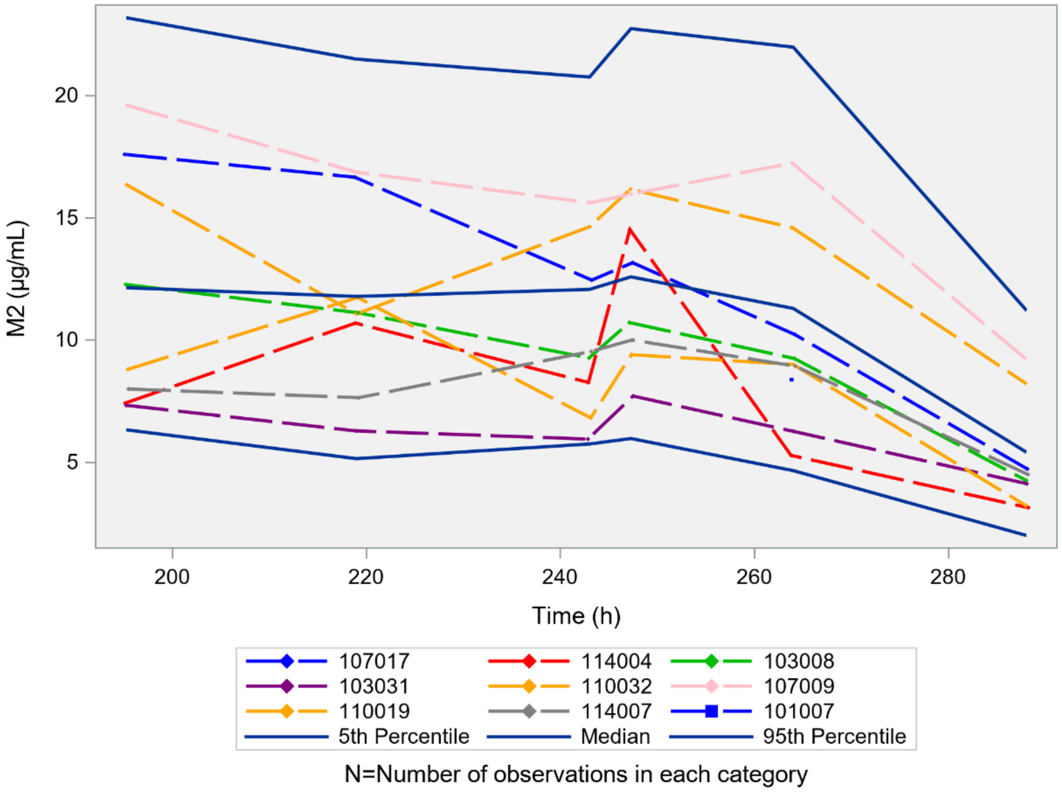

The 9 patients with confirmed g-HAT relapse at 18 months were included in the analysis (the patient who died from causes unrelated to fexinidazole and the 2 patients who were lost to follow-up were not considered).
M1= fexinidazole sulfoxide; M2=fexinidazole sulfone; PK=pharmacokinetic.
