# Supplementary material for: Effectiveness and safety of fexinidazole for gambiense human African trypanosomiasis and exploration of adherence in outpatients: a phase 3b, prospective, open-label, non-randomised, cohort study
Source: Lancet Glob Health. 2025 Apr 24;13(5):e900–9. doi: 10.1016/S2214-109X(24)00526-6 (PMC12041186; doi:10.1016/S2214-109X(24)00526-6)
Supplement: Equitable Partnership Declaration [file mmc3.pdf]

THE LANCET
Global Health

Supplementary appendix 3

This Equitable Partnership Declaration (EPD) was submitted by the authors, and we reproduce it as supplied. It has not been peer reviewed. *The Lancet*'s editorial processes have not been applied to the EPD.

Supplement to: Kumeso VKB, Perdrieu C, Menétrey C, et al. Effectiveness and safety of fexinidazole for *gambiense* human African trypanosomiasis and exploration of adherence in outpatients: a phase 3b, prospective, open-label, non-randomised, cohort study. *Lancet Glob Health* 2025; **13:** e900–09.

Equitable Partnership Declaration

Researcher considerations

1. Please detail the involvement that researchers who are based in the region(s) of study had during a) study design; b) clinical study processes, such as processing blood samples, prescribing medication, or patient recruitment; c) data interpretation; and d) manuscript preparation, commenting on all aspects. If they were not involved in any of these aspects, please explain why.

   This question is intended for international partnerships; if all your authors are based in the area of study, this question is not applicable.

   This should include a thorough description of their leadership role(s) in the study. Are local researchers named in the author list or the acknowledgements, or are they not mentioned at all (and, if not, why)? Please also describe the involvement of early career researchers based in the location of the study. Some of this information might be repeated from the Contributors section in the manuscript. Note: we adhere to [ICMJE authorship criteria] when deciding who should be named on a paper.

**a) Study design:** This study builds on several previous clinical trials done in collaboration with the Ministries of Health (MoH) Human African Trypanosomiasis Control Programmes (PNLTHA in their French acronym) of the Democratic Republic of Congo (DRC) and Guinea. Most local researchers, engaged through the MoH, had participated in previous trials and had an essential role in identifying the trial sites and their needs of equipment, as well as modulating the protocol content towards the field realities. A coordinating team, led by an overall trial investigator from the DRC, was set up in Kinshasa and participated in the preparation of the study protocol. The design of the study procedures followed the previous experience and the national guidance for treatment, follow-up, and surveillance, coupled with specific information required for the trial by the sponsor at Drugs for Neglected Diseases initiative (DNDi) and the industrial partner Foundation S (including specific assessment of the understanding of trial instructions by the participants treated at home and by their caretakers.)
**b) Clinical study processes:** The trial procedures were defined and timed in direct consultations with the local coordination team and the investigators, most of whom had already participated in the previous studies. Each trial site kept a trial master file locally. Coordination of monitoring and technical supervision was undertaken by a specific local team of DNDi based in Kinshasa, with occasional support from the team based in Europe. Participant selection was entirely done by the investigators, from cases identified through the normal case detection processes of the PNLTHA of the DRC and Guinea. All study procedures were done by the local investigators and their teams. Most laboratory and medical exams were performed at each trial site, where laboratories had been upgraded to be able to conduct the study tests. Only pharmacokinetics of the study drug and ECG specialist analysis were done in Europe. ECGs were initially examined by the investigators regarding the safety of individual participants.
**c) Data interpretation:** During the trial, medical and general monitoring allowed for regular exchanges among local researchers and also between Congolese researchers, Guinean researchers, and the European management and support team to clarify and clean the data. Preparation and implementation of the statistical analysis plan was sub-contracted externally to a French company. Discussions were held with the Congolese and Guinean investigators, either individually on concrete cases or collectively at several investigator's meetings, including a review of the full results before preparing the study report. Clinical relevance of the findings was determined by the local investigators from each trial site.

**d) Manuscript preparation:** The manuscript draft was prepared by a medical writer, based on the larger study report, and reviewed by all co-authors who were free to add comments or propose changes in the text.

2. Were the data used in your study collected by authors named on the paper, or have they been extracted from a source such as a national survey? ie, is this a secondary analysis of data that were not collected by the authors of this paper. If the authors of this paper were not involved in data collection, how were data interpreted with sufficient contextual knowledge?

   The Lancet Global Health *believe contextual understanding is crucial for informed data analysis and interpretation.*

In the list of authors, the first one (VKBK) was the overall study senior principal investigator based in Kinshasa. The third, counting from the last one, was the coordinating investigator (WMK), who was supported by n°4 (MIWK), and n°5 (DNT) in the DRC and by n°6 (MC) in Guinea. They ensured harmonisation of the interpretation from the site investigators, who are from n° 7 to n°18 (JT, PK, MLC, AK, WKM, FAM, HM, JM, TMM, GM, DMMN, SLN). Except MLC and AK from Guinea, all the others were from the DRC. They were the team leader from their hospitals (trial sites) who gathered all data. All of them were leading clinicians from their respective hospitals.

3. How was funding used to remunerate and enhance the skills of researchers and institutions based in the area(s) of study? And how was funding used to improve research infrastructure in the area of study?

   Potentially effective investments into long-term skills and opportunities within institutions could include training or mentorship in analytical techniques and manuscript writing, opportunities to lead all or specific aspects of the study, financial remuneration rather than requiring volunteers, and other professional development and educational opportunities.

   Improvements to research infrastructure could be funding of extended trial designs (such as platform trials) and use of master protocols to enable these designs, establishment of long-term contracts for research staff, building research facilities, and local control of funding allocation.

**Skills:** Even if most investigators had already participated in previous trials with the same product, specific training sessions, both in good clinical practice and study procedures were completed at the trial initiation visits at each site. Regular contacts and visits to the sites by the coordinating team from Kinshasa allowed to address any doubts, exchange new information or train new staff of each site trial team. Training targeted specifically any new procedure that was not performed routinely by the clinical teams before the trial.
**Research infrastructure:** As the health system at the DRC and Guinea remains precarious, the project had to invest to create adequate laboratories to fulfill the study data needs, especially when they were specific to the study. Most of this rehabilitation and preparation was done with previous trials, in this case only maintenance and upgrading were needed, including adequate waste management and solar energy allowing for an autonomous supply, as the general electricity supply from the government was often poorly functional (if existing). Special attention was dedicated to the purchase of a solar cold chain including fridges for some laboratory consumables.

Patient infrastructure had been renewed, including beds, mattresses, cupboards, other furniture and equipment and a general upgrade of the rooms for the patients and staff. Internet was set up in every trial site so that information could be shared rapidly, especially in case of serious adverse events.

4. How did you safeguard the researchers who implemented the study?

Please describe how you guaranteed safe working conditions for study staff, including provision of appropriate personal protective equipment, protection from violence, and prevention of overworking.

Special training followed by direct on-site supervision was organised on standard precautions, hygiene and waste management including preparation of safe closed waste areas, digging new waste pits allowing for waste separation and new or renewed incinerators for each hospital. Routine protective equipment was provided from the start, reinforced with additional equipment in April 2020 at the beginning of the COVID-19 pandemic, during the final stages of the study follow-up visits. Risk of communal violence was monitored in the DRC, although low-risk trial sites had been selected considering also security factors. The study coordinator could choose not to include a patient if he/she felt that this would lead to overwork, but in general, human African trypanosomiasis had been reducing the number of detected cases over time and this situation did not happen. A specific emergency treatment backpack from Médecins Sans Frontières (MSF) Logistique was provided to all sites, in principle to treat the patients for possible study safety issues, but the staff could also use it if needed without restrictions.

Benefits to the communities and regions of study

5. How does the study address the research and policy priorities of its location?

How were the local priorities determined and then used to inform the research question? Who decided which priorities to take forward? Which elements of the study address those priorities?

At the start of the development of fexinidazole in previous trials, the need for a new drug was overwhelming due to the long-lasting neglect that sleeping sickness had had until recently, with existing drugs either toxic, or difficult to administer. The study sites were selected according to their higher disease prevalence, based on the data of the National Sleeping Sickness Control Programmes. The study procedures, including food for the patients, were set in place according to each hospital situation which was carefully assessed by a multidisciplinary team. Transport of patients was funded due to their additional cost of travelling from their homes. All those were procured at no cost, including treatment and care for concomitant diseases or pregnancies that could be needed during the study participation.

6. How will research products be shared in the community of study?

For instance, will you be providing written or oral layperson summaries for non-academic information sharing? Will study data be made available to institutions in the region(s) of study? The Lancet Global Health *encourages authors to translate the summary (abstract) into relevant languages after paper editing; do you intend to translate your summary?*

Thanks to the previous trials, of which this one was an extension, all affected communities are nowadays benefiting from the new approved treatment with fexinidazole. Recent information from the WHO reported that fexinidazole is being administered to over 70% of all identified patients of gambiense sleeping sickness in all sub-Saharan African endemic countries. Information on the use, efficacy and safety of the product have been extensively shared during a parallel access to treatment project, targeting 5 countries, emphasizing the DRC, which is the most endemic. Study data have been shared locally through two main tools: presentations at scientific meetings in African countries (ISCTRC or HAT Platform conferences) and the HAT Platform Newsletter. Additional information on the HAT Platform as well as electronic versions of all newsletters can be found here: HAT Platform | DNDi

7. How were individuals, communities, and environments protected from harm?

a) *How did you ensure that sensitive patient data was handled safely and respectfully? Was there any potential for stigma or discrimination against participants arising from any of the procedures or outcomes of the study?*

Previous clinical trials had already shown the acceptable efficacy and safety of fexinidazole. The product was registered in the DRC before completing this trial. All patients had an Identification number whose full reference coordinates remained strictly confidential and kept by each investigator in their own local trial documentation. Human African trypanosomiasis usually leads to stigma because of the disease symptoms, but once treated the stigmatisation was reduced. Trial procedures were undertaken at the hospital and were not very different from the usual procedures. HIV was not tested in the study.

b) *Might any of the tests be experienced as invasive or culturally insensitive?*

Initial diagnosis and follow-up assessment required lumbar puncture, which is perceived as invasive, but the study just followed the procedures requested by the national control programmes in their routine surveillance. Other tests or physical examination were of no special significance: ECG, haematology, biochemistry, parasitology, pharmacokinetics dry blood spot.

c) *How did you determine that work was sensitive to traditions, restrictions, and considerations of all cultural and religious groups in the study population?*

After showing safety and efficacy in previous trials, pregnant (from the 2nd trimester) and breastfeeding women were eligible to participate, thus rules for contraception were light, knowing that the subject remains socially contentious in the DRC (especially among husbands). In parallel to this study, an ethnographic survey was completed on the community perceptions about the disease, its transmission, health seeking behaviour and treatment. Before the trial, a careful review of social sciences research in the DRC was also completed.

d) *Were biowaste and radioactive waste disposed of in accordance with local laws?*

No radioactive waste was generated, biowaste was disposed according to the newly built protected waste area, including a specific pit for biologically contaminated was separated from sharps, glass or common waste. Incineration capacity was upgraded in all sites. Specific training on waste separation was done from the beginning of the trial and followed by supervisory visits.

e) *Were any structures built that would have impacted members of the community or the environment (such as handwashing facilities in a public space)? If so, how did you ensure that you had appropriate community buy-in?*

The modifications of existing structures consisted in improving hospital capacities and did not have any impact at the community level other that improved services and hygiene. Specific rest

and cooking areas have also been built within the hospital premises, as well as latrines for the patients.

f) *How might the study have impacted existing health-care resources (such as staff workloads, use of equipment that is typically employed elsewhere, or reallocation of public funds)?*

There were no complaints regarding added workload as those patients would have been cared for at the trial site anyway. Most needed equipment and all consumables had been provided by the study, and those that would be useful after the end of the study were donated to the trial sites. All trial costs including economic compensation to the staff, food for the patients were provided and hospital equipment and drugs from the hospital pharmacy for concomitant diseases were paid for their use.

8. Finally, please provide the title (eg, Dr/Prof, Mr/Mrs/Ms/Mx), name, and email address of an author who can be contacted about this statement. This can be the corresponding author.

Name: Dr Olaf Valverde Mordt
Email: ovalverde@dndi.org
